# Increased glutamate transporter-associated anion currents cause glial apoptosis in episodic ataxia 6

**DOI:** 10.1093/braincomms/fcaa022

**Published:** 2020-03-04

**Authors:** Peter Kovermann, Verena Untiet, Yulia Kolobkova, Miriam Engels, Stephan Baader, Karl Schilling, Christoph Fahlke

**Affiliations:** f1 Institut für Biologische Informationsprozesse, Molekular- und Zellphysiologie (IBI-1), Forschungszentrum Jülich, 52428 Jülich, Germany; f2 Anatomisches Institut, Anatomie und Zellbiologie, Rheinische Friedrich-Wilhelm Universität Bonn, 53115 Bonn, Germany

**Keywords:** glutamate transporters, chloride homeostasis, Bergmann glia, glial apoptosis, episodic ataxia

## Abstract

Episodic ataxia type 6 is an inherited neurological condition characterized by combined ataxia and epilepsy. A severe form of this disease with episodes combining ataxia, epilepsy and hemiplegia was recently associated with a proline to arginine substitution at position 290 of the excitatory amino acid transporter 1 in a heterozygous patient. The excitatory amino acid transporter 1 is the predominant glial glutamate transporter in the cerebellum. However, this glutamate transporter also functions as an anion channel and earlier work in heterologous expression systems demonstrated that the mutation impairs the glutamate transport rate, while increasing channel activity. To understand how these changes cause ataxia, we developed a constitutive transgenic mouse model. Transgenic mice display epilepsy, ataxia and cerebellar atrophy and, thus, closely resemble the human disease. We observed increased glutamate-activated chloride efflux in Bergmann glia that triggers the apoptosis of these cells during infancy. The loss of Bergmann glia results in reduced glutamate uptake and impaired neural network formation in the cerebellar cortex. This study shows how gain-of-function of glutamate transporter-associated anion channels causes ataxia through modifying cerebellar development.

## Teaser

Episodic ataxia type 6 is a genetic disease combining ataxia and epilepsy that is caused by dysfunctional glutamate transporters. Kovermann *et al.* studied a transgenic animal model and found that transporter dysfunction induces ataxia by triggering glial apoptosis via disturbed chloride homeostasis, resulting in reduced glutamate uptake and cerebellar degeneration.

## Graphical Abstract

**Graphical Abstract:**
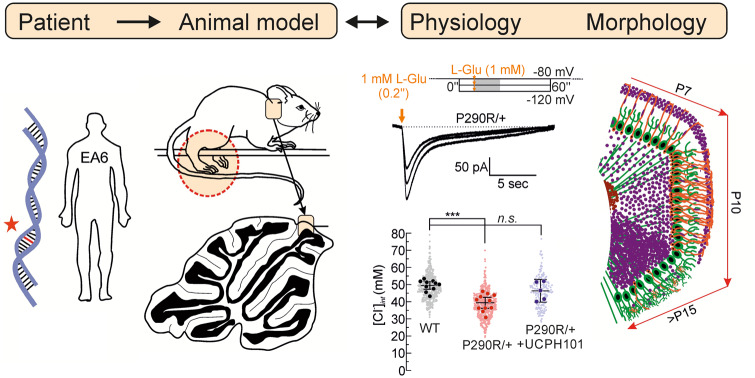

## Introduction

Episodic ataxias (EAs) are rare neurological syndromes characterized by paroxysmal cerebellar incoordination, variably combined with epilepsy, dystonia and migraine-like headache (Jen *et al.*, 2007). Eight variants of autosomal dominant EAs (EA1–8) have been defined with different genetic origins. EA6 has been reported in only seven families, with clinical symptoms differing from those of other EA forms by the addition of long-lasting attacks of ataxia and epilepsy and the absence of myokymia, nystagmus and tinnitus. Mutations in *SLC1A3*, encoding the glial excitatory amino acid transporter 1 (EAAT1), the human homologue of the glutamate–aspartate transporter (GLAST) (Storck *et al.*, 1992), were identified in all seven families (Jen *et al.*, 2005; de Vries *et al.*, 2009; Pyle *et al.*, 2015; Choi *et al.*, 2017a, *b*; Iwama *et al*., 2018). 

A heterozygous *SLC1A3* missense mutation predicting arginine replacement of a proline residue in transmembrane domain 5 of *h*EAAT1 (P290R) was identified in a 10-year-old boy, who had suffered from episodes of ataxia, epilepsy and hemiplegia throughout his childhood (Jen *et al.*, 2005). The patient also experienced attacks of headache, visual field neglect and hemiplegia for at least twice the duration of those in other EA patients. Magnetic resonance imaging scan showed mild cerebellar atrophy, and electroencephalography revealed subclinical seizure activity in one episode and epileptiform discharges in another one (Jen *et al.*, 2005). EAATs function as both secondary-active glutamate transporters and anion channels (Fahlke *et al.*, 2016) and P290R substitution exerts opposing effects on these functions in heterologous expression systems. It reduces the speed of conformational changes associated with Na^+^ association/dissociation with the outward-facing EAAT1, thereby decreasing the glutamate transport rate and increasing the open probability of the anion channel (Winter *et al.*, 2012; Hotzy *et al.*, 2013).

To investigate how changes in glial glutamate transporter function cause the neurological symptoms of EA6, we used a constitutive heterozygous mouse model carrying the targeted missense mutation in EAAT1/GLAST (*Slc1a3^P290R/+^*).

## Materials and methods

### Animals

Wild-type (WT), homozygous GLAST knock-out (*Slc1a3*^−^^/^^−^) and heterozygous GLAST mutant (*Slc1a3^P290R/+^*) mice of both sexes with the genetic backgrounds, C57BL/6N (WT, *Slc1a3^P290R/+^*) and 129/SvJ (WT, *Slc1a3*^−/−^, *Slc1a3^P290R/+^*) and FVB/N [Tg(GFAP–EGFP)14Mes/J], were studied at ages between P5 and P800 (Supplementary Material). Mouse studies in this study conform with the Animal Research: Reporting of *In Vivo* Experiments guidelines (McGrath *et al.*, 2010).

### Motor coordination testing

Motor coordination was tested in the ledge test (Guyenet *et al.*, 2010) by manually placing WT and mutant mice on a 1-cm wide ledge and encouraging them to walk along the ledge for 2′ by gentle nudges (Supplementary Material). Rotarod testing was performed by placing female mice (∼P50) on an accelerating rotarod (Ugo-Basile, Italy) and measuring latencies to fall off the rotating rod. Mice usually underwent a short training and were subjected to three test sessions within two consecutive days (Day 1, 10:00–12:00 h and 14:00–16:00 h; Day 2, 10:00–12:00 h) after the successful completion of this training. Each session consisted of three trials 10′ apart (Supplementary Material).

### Visualization and quantification of specific cell types and components

Details about staining procedures and used antibodies are provided in the Supplementary Material and in Supplementary Table 2.

### Preparation of acute cerebellar slices

After anesthetizing animals (P7–P900) with isoflurane and rapid decapitation, brains were placed in ice-cold oxygenated Ringer’s solution A (125 mM NaCl, 2.5 mM KCl, 1.25 mM NaH_2_PO_4_, 26 mM NaHCO_3_, 0.5 mM CaCl_2_, 5 mM MgCl_2_, 20 mM C_6_H_12_O_6_, 5% CO_2_ and 95% O_2_). Sagittal cerebellar slices (250-µm thick) were cut using a microtome (*υ* = 60 Hz, amplitude = 1 mm) and transferred to a gauze slice holder in oxygenated Ringer’s solution A for 30′ at 37°C and 90′ to Ringer’s solution B (125 mM NaCl, 2.5 mM KCl, 1.25 mM NaH_2_PO_4_, 26 mM NaHCO_3_, 2 mM CaCl_2_, 1 mM MgCl_2_ and 25 mM C_6_H_12_O_6_) at room temperature (RT). During experiments, the slices were constantly perfused with oxygenated Ringer’s solution B or oxygenated artificial cerebrospinal fluid (125 mM NaCl, 2.5 mM KCl, 1.25 mM NaH_2_PO_4_, 26 mM NaHCO_3_, 2 mM CaCl_2_, 1 mM MgCl_2_). All experiments with acute slices were completed within 8 h after brain removal.

### Cl^−^ current recordings in Bergmann glia cells

We performed whole-cell recordings of Bergmann glia cell anion currents in acute cerebellar slices of mice between P9 and P14 under continuous perfusion with artificial cerebrospinal fluid at RT supplemented with 1 µM 6-cyano-7-nitroquinoxaline-2,3-dione (Tocris Bioscience, Germany) using an EPC10 USB amplifier with PatchMaster software (HEKA Elektronik). Bergmann glia cells were visually identified by morphology and location in the Purkinje neuron layer. The pipette solution contained 145 mM KCl, 1 mM MgCl_2_ and 10 mM HEPES/KOH (pH 7.2). Cells were held at membrane potentials of −80/−100/−120 mV, and glutamate-activated anion currents were elicited by the application of Na glutamate (1 mM) by a pressure-driven perfusion system (PDES-DXH; NPI Electronic GmbH, Germany) coupled to standard micropipettes (Hilgenberg, Germany) placed 10–20 µm from the cell somata. A maximum of one Bergmann glia cell was tested per slice.

### Fluorescence lifetime imaging microscopy

We measured the internal chloride concentration ([Cl^**−**^]_int_) of Bergmann glia cells by 1-(ethoxycarbonylmethyl)-6-methoxy-quinolinium bromide (MQAE) fluorescence lifetime imaging microscopy with an upright fluorescence microscope (A1 MP, Nikon, Netherlands) equipped with a 25× water immersion objective (Gensch *et al.*, 2015; Untiet *et al.*, 2017). Acute cerebellar slices were incubated with 3.5 mM MQAE in Ringer’s solution B for 30′. MQAE fluorescence is collisional quenched by Cl^−^ ions, resulting in a linear relationship between the inverse fluorescence lifetime and [Cl^**−**^]_int_ (Verkman, 1990):

(1) $$\frac{\tau_{0}}{\tau }=1+K_{\mathrm{SV}}\;[{\mathrm{Cl}}^{-}{]}_{\mathrm{int},}$$

where *τ* is the MQAE fluorescence lifetime at a given [Cl^−^]_int_, *τ*_0_ is the MQAE fluorescence lifetime in the absence of chloride and *K*_SV_ is the cell type-specific Stern–Volmer constant. After calibration in Bergmann glia cells using the 2-ionophore calibration experiments (Untiet *et al.*, 2017), [Cl^**−**^]_int_ for single-cell soma was calculated from the mean fluorescence lifetime of all pixels within a defined region of interest. [Cl^**−**^]_int_ are given as mean ± 95% confidence interval (CI) from 3–4 individual animals per time point and genotype.

### Apoptosis assays

Bergmann glia cells were identified in acute cerebellar slices from the progeny of GFAP-conjugated enhanced green fluorescent protein (GFAP–EGFP) reporter mice [FVB/N(GFAP–EGFP)14MES/J] crossbred with *Slc1a3^P290R/+^* mutants. After anesthetizing animals with isoflurane and rapid decapitation, brains were transferred into 4% paraformaldehyde in phosphate buffer (30′, 4°C). Cerebella were then washed in phosphate buffer (30′, RT) and incubated in phosphate buffer supplemented with 10% C_12_H_22_O_11_ (60′, RT) and 30% C_12_H_22_O_11_ (12 h, 4°C) before embedding in NEG50 (Thermo Fisher Scientific, USA) and cut into sagittal slices (18 µm thick), using a HM560 microtome (MICROM, Germany). Slices were incubated with antibody against rabbit anti-active caspase 3 (CASP3) to label apoptotic cells. For immunochemical analysis, slices were incubated in CTA (5% ChemiBLOCKER—Merck-Millipore, 0.5% TritonX-100—Sigma Aldrich, 0.05% NaN_3_, v/v) for 10′ and then overnight in CTA with primary antibody at RT. Secondary antibodies were applied in CTA for 60′. Apoptosis was quantified by averaging the number of CASP3-positive Bergmann glia cells per brain slice for each tested animal. Terminal dUTP nick-end labelling (TUNEL) of fragmented DNA in cerebellar nuclei was performed as a control for Bergmann glial apoptosis as described in Supplementary Material. Details about used antibodies/kits are listed in Supplementary Table 2.

### Cell‐attached recordings from Purkinje neurons

We visually identified Purkinje neurons in acute brain slices based on size and location between the granule cell layer and the molecular layer. For electrophysiological recordings, pipettes (4–6 MΩ) were filled with a Ringer-like solution (140 mM NaCl, 4 mM KCl and 10 mM HEPES/KOH, pH 7.4) and slices were constantly perfused with oxygenated artificial cerebrospinal fluid (125 mM NaCl, 2.5 mM KCl, 2 mM CaCl_2_, 2 mM MgCl_2_, 1.25 mM NaH_2_PO_4_ and 26 mM NaHCO_3_) during the course of the experiment. Cell-attached patches were formed at neuron somata, and currents were recorded at RT between 30'' and 10' in voltage‐clamp mode at 0 mV (EPC10 amplifier; HEKA Elektronik). Data were analysed offline using Clampfit event-analysis functions (Molecular Devices, USA).

### Statistical analysis

All statistical parameters were calculated with SigmaPlot (Systat Software GmbH), OriginPro (OriginLab Corp.) or Excel (Microsoft Corp.). Data are presented as means (*x̄*), medians (*x˜*_0.5_) ± CI (95% confidence interval) or *σ* (standard deviation) from individual animals. Ages are provided as postnatal days. Data were analysed using Mann–Whitney *U*-tests or two-way ANOVA tests and Kruskal–Wallis ANOVA on ranks with Holm–Sidak or Dunn’s *post hoc* testing. *P***-**values ≤0.05 were considered statistically significant with *P ≤* *0.05, **P ≤* *0.01, ***P ≤* *0.001; all *P*-values are provided in Supplementary Table 3.

### Data availability

The source data that support the findings of this study are available from the corresponding author upon reasonable request.

## Results

### *Slc1a3^P290R/+^* mice suffer from epilepsy and ataxia

**Figure 1:**
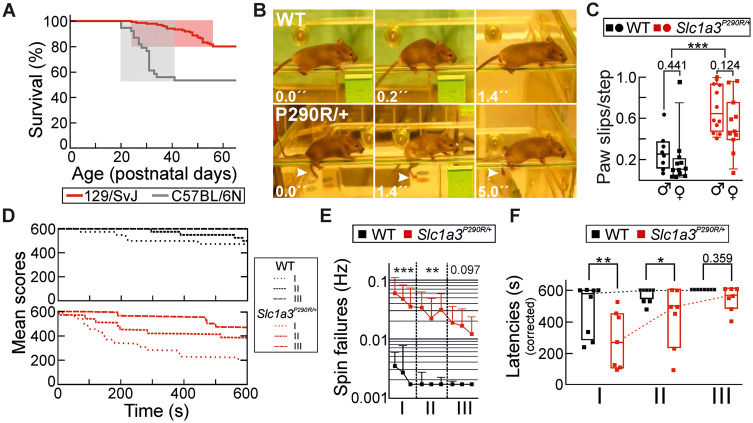
***Slc1a3^P290R/+^* mice suffer from epilepsy and ataxia.** Image **A** depicts survival rates for C57BL/6 (grey) and 129/SvJ (red) *Slc1a3^P290R/+^* mice over time, with shaded areas indicating periods with pronounced seizure activity. Representative screen shots (**B**) from video recordings of WT and *Slc1a3^P290R/+^* mice moving on a ledge indicate that transgenic mice tend to slip (arrowheads) from the ledge with their hindlimbs. The whisker plot (**C**) shows the median paw slip numbers per step for adult (P50) WT (black) or *Slc1a3^P290R/+^* (red) mice of both sexes (circles, ♂; squares, ♀). The analysis revealed no detectable sex differences in Ledgetest performance (*P *=* *0.110) but pronounced differences between genotypes in both sexes (*n *=* *8/12/12/11, WT^♂^/WT^♀^/Mut^♂^/Mut^♀^, *P *≤* *0.001^♂^/*P *=* *0.001^♀^). The averaged time series of mean scores (**D**) for three consecutive rotarod trial sessions (I–III) with adult female WT (black) or *Slc1a3^P290R/+^* (red) mice at P50 point to differences between WT and mutant animals. The increased mean spin failure frequencies (**E**) for WT or *Slc1a3^P290R/+^* (*P *=* *0.011) and the decreased rotarod latencies to fall (**F**) indicate that *Slc1a3^P290R/+^* mice suffer from ataxia (*n *=* *8/7, WT/Mut, *P *=* *0.031). Data are shown as whisker-box plots with medians [whisker: 5th to 95th percentile, box: 25th to 75th percentiles, single (**C**) and repeated measure (**E** and **F**) two-way ANOVA and Holm–Sidak *post hoc* test]; *P ≤* *0.05, **P ≤* *0.01, ***P ≤* *0.001; all *P*-values for this figure are provided in Supplementary Table 3.

Knock-in *Slc1a3^P290R/+^* mice (*Slc1a3^tm1P290RCfa^*) were initially generated by homologous recombination in the C57BL/6N background (Supplementary Material and Supplementary Fig. 1). Heterozygous mice suffered from spontaneous generalized seizures and death during the weaning period with a peak between postnatal days (P) P25– and P30 (Fig. 1A, grey), and we therefore backcrossed the mutation into the 129/SvJ strain, as a similar procedure has been successfully used to generate another animal model of severe epilepsy (Yu *et al.*, 2006). This change in genetic background reduced lethal seizure activity by ∼70% (*n *=* *39/150, C57/129 Sv) and delayed the onset of the seizure period and the peak time of premature deaths to P35**–**P55 (Fig. 1A, red and Supplementary Video 1). We monitored WT and *Slc1a3^P290R/+^* mice and scored fitness with respect to general condition, behavioural aspects and EA6 associated pathology (Supplementary Table 1). Mutant male mice exhibited a severe phenotype with maximum scores between P40 and P80, whereas female *Slc1a3^P290R/+^* mice showed a milder constitutive phenotype (Supplementary Fig. 2).

We assessed motor coordination by observing the *Slc1a3^P290R/+^* animals walking along the ledge of a cage (Fig. 1B) (Guyenet *et al.*, 2010). Ledge tests showed significantly more paw slips per step number in mutant animals (Fig. 1C and Supplementary Video 2), with no difference between sexes (*n *=* *8/12/12/11, WT^♂^/WT^♀^/Mut^♂^/Mut^♀^). Gait coordination was additionally tested by measuring the time animals can walk on a rotating horizontal rod (Jones and Roberts, 1968) (Fig. 1D–F). Figure 1D shows averaged time series of consecutive rotarod sessions (labelled I–III) for WT and mutant animals on Kaplan–Meier curves (Kaplan and Meier, 1958). These values were corrected for episodes, in which mice did not walk forward, but rotated with the rotarod by holding on to it (spin failures, Supplementary Material; Fig. 1E and Supplementary Fig. 3A). In initial tests, we observed a much higher frequency of such episodes for mutant male than animals for mutant female animals and, therefore, restricted rotarod testing to female animals (Supplementary Material). Compared with WT mice, mutant mice remained shorter times on the rotating rod and started significantly earlier to make mistakes (*n *=* *8/7 animals, WT/Mut, Fig. 1F and Supplementary Fig. 3B). In the third trial, all WT performed the task without falling off and without spin failures, whereas 4/7 *Slc1a3^P290R/+^* animals still either fall off or rotated with the waltz (Fig. 1D–F and Supplementary Fig. 3). In conclusion, *Slc1a3^P290R/+^* animals exhibit a robust EA6 phenotype with epilepsy and ataxia, thus resembling the phenotype of humans carrying the same mutation.

### Cerebellar Bergmann glia cell*s* from *Slc1a3^P290R/+^* mice degenerate during the second and third postnatal weeks

**Figure 2:**
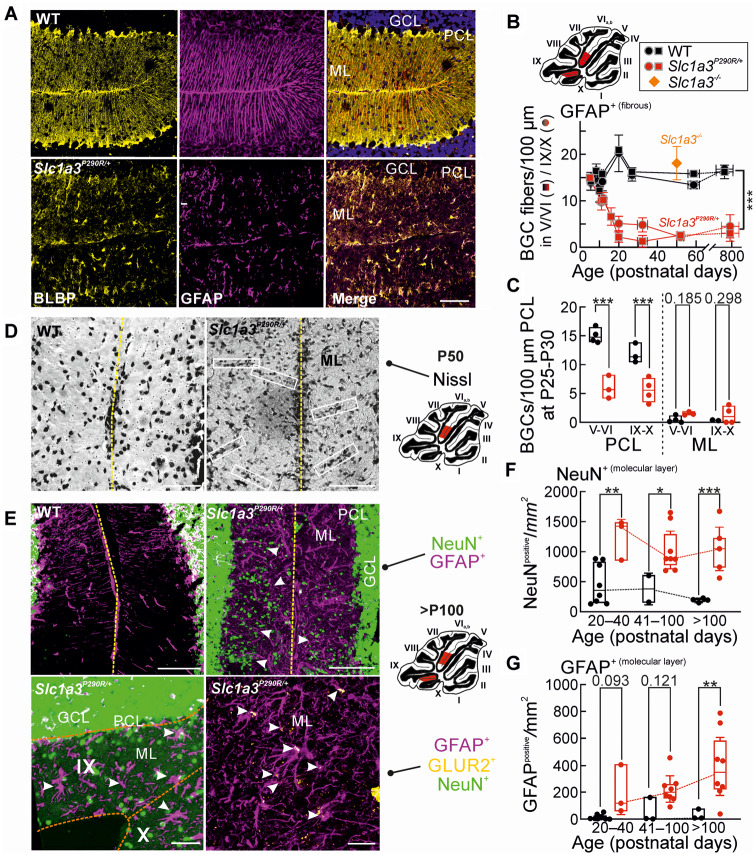
**Degeneration of Bergmann glia cells and cellular reorganization of the cerebellar cortex in *Slc1a3^P290R/+^* mice.** Representative confocal images (**A**) of paraffin-embedded cerebellar WT (*top*) or *Slc1a3^P290R/+^* (*bottom*) slices show the vermis region of lobe V/VI immunostained for BLBP (yellow) and GFAP (P20, magenta) and indicate age-dependent changes in the numbers of Bergmann glia cell fibres (**B**) in the MLs of WT (black), *Slc1a3*^−/−^ (orange) or *Slc1a3^P290R/+^* (red) cerebella. The data represent mean numbers of GFAP^positive^ fibres per 100 µm PCL in individual animals (*n *=* *25/32 animals, WT/Mut). Bergmann glial cell numbers were counted (**C**) in the PCL and in the ML and indicated that the median cell loss is much higher than the number of ectopic cells in the ML. Cerebellar regions stained for Nissl (**D**, P50) or stained for NeuN (**E**, >P100, green) indicate regions with anomalous enrichment of NeuN^positive^ cells (**E**, arrowheads). The white boxes depict linear arrangements of Nissl-stained nuclei within the ML of *Slc1a3^P290R/+^* mice (**D**). The whisker-box plots (**F** and **G**) show increased density of NeuN^positive^ cells (F, *n *=* *14/16 animals, WT/Mut) and increased density of GFAP^positive^ cells (G, *n *=* *14/19 animals, WT/Mut) in mutant lobes V/VI. Astrocytes mislocalize to the ML in mutant cerebella; confocal images of the nodular region (**E**, *bottom*) of an *Slc1a3^P290R/+^* animal (>P100) immunostained for GFAP (magenta) and NeuN (green) indicate multipolar astrocytes in the ML from mutant animals (**E**, *bottom*, left). Colocalization (white) of GLUR2 (yellow) with GFAP (magenta) in the ML (**E**, *bottom*, right) of an *Slc1a3^P290R/+^* mouse shows that GFAP^positive^ multipolar astrocytes do not emerge from the Bergmann glia cell pool (see also Supplementary Fig. 6). The density of GFAP^positive^ multipolar cells in the MLs increases over time in *Slc1a3^P290R/+^* mice but not in WT mice. Scale bars: 100 µm (**A**), 50 µm (**D**) and 50/25 µm (**E**, *top*/*bottom*). Numbers of Bergmann glia fibres over time (**B**) are shown as means ± SEM of individual animals, and genotype-specific differences over time were analysed with two-way ANOVA and Holm–Sidak *post hoc* testing. Data in **C**, **F** and **G** are shown as whisker-box plots (whisker: ±CI, box: 25th to 75th percentiles with each data point representing the values obtained from one individual animal; two-way ANOVA and Holm–Sidak *post hoc* tests); *P ≤* *0.05, **P ≤* *0.01 and ***P ≤* *0.001; all *P*-values for this figure are provided in Supplementary Table 3. GCL, granule cell layer; ML, molecular layer; PCL, Purkinje cell layer.

As EAAT1/GLAST is highly expressed in cerebellar Bergmann glia cells (Rothstein *et al.*, 1994; Torp *et al.*, 1994; Chaudhry *et al.*, 1995; Watase *et al.*, 1998), alterations in these cells likely represent initial steps in cerebellar dysfunction in *Slc1a3^P290R/+^* mice. Figure 2A shows representative confocal images from the cerebellar cortex of WT and mutant mice (P20), immunostained with antibodies against glial fibrillary acidic protein (GFAP) and against brain lipid-binding protein (BLBP). Anti-BLBP permits the visualization of Bergmann glia soma (Feng *et al.*, 1994), whereas anti-GFAP stains Bergmann glia fibres. In WT animals, Bergmann glia cells with typical unipolar morphology, i.e. soma in the Purkinje neuron layer and extensions in the molecular layer, were readily observed; however, such structures were absent in *Slc1a3^P290R/+^* mice at P20. We identified Bergmann glia cell fibres as GFAP-positive processes in the cerebellar molecular layer, which originate from Purkinje neuron layers, and counted them in WT and mutant animals between P5 and P800. Prior to P10, WT and mutant animals had similar fibre numbers, indicating the normal development of Bergmann glia in *Slc1a3^P290R/+^* mice to this age. However, between P10 and P20, a pronounced age-dependent reduction in Bergmann glia cell fibre numbers occurred in *Slc1a3^P290R/+^* cerebella (Fig. 2B). GFAP-positive Bergmann glia fibres were reduced in all cerebellar regions, while glial development remained unaffected in the hippocampus and the cerebral cortex (data not shown). The number of Bergmann glia cell soma and fibres was reduced by a similar percentage (Fig. 2C), indicating loss of Bergmann glia cells and not mere retraction of fibres in the *Slc1a3^P290R/+^* cerebellum. Whereas the Bergmann glial cells were reduced by >70% in the Purkinje layer of mutant animals, the number of glial cells only slightly increased in the molecular layer (Fig. 2C). Immunostaining of cerebellar slices and western blotting of cerebellar lysates against the Bergmann glia markers BLBP, S100β and GLAST showed decreased numbers of Bergmann glia cell somata (Supplementary Fig. 4A and B) and reduced relative protein expression of the markers (Supplementary Fig. 4C and D and Supplementary Material). These results indicate Bergmann glia cell death rather than mislocalization of glial cells to the molecular layer. In *Slc1a3*^−/−^ (*Slc1a3^tm1Kta^*, GLAST knock-out) mice, the density of Bergmann glia cell fibres was not different from WT at P50 (Fig. 2B), demonstrating that loss of EAAT1/GLAST glutamate transporter does not result in Bergmann glia cell degeneration at this age (Watase *et al.*, 1998).

### Altered cell distribution in the cerebellar cortex of *Slc1a3^P290R/+^* mice

*Slc1a3^P290R/+^* animals vary widely in cerebellar morphology, ranging from increased density of granule cells in the molecular layer (Fig. 2D–F and Supplementary Fig. 5A–F) to massive degeneration associated with foamy syncytial structures (Supplementary Fig. 5G and H). In contrast, adult WT cerebella are characterized by a defined layered structure with virtually no granule cells in the molecular layer. Since fibres from Bergmann glia cells and from their progenitors serve as migration pathways for cerebellar granule cells in the developing cerebellum (Rakic, 1971; Hatten *et al.*, 1984), changes in Bergmann glia function might result in the mislocalization of granule cells. In mutant cerebella without severe degeneration, we regularly observed mislocalized cells that are recognized by antibodies against NeuN, a specific nuclear marker for cerebellar granule cells (Mullen *et al.*, 1992; Weyer and Schilling, 2003) (Fig. 2D and E). We conclude that mutant EAAT1/GLAST expression impairs granule cell migration to the internal granule cell layer during development.

We also observed multipolar astrocytes in the molecular layer of *Slc1a3^P290R/+^* cerebella, which did not only differ from Bergmann glia cells in localization of their cell somas but also in morphology (Fig. 2E and G). We co-stained slices with antibodies against glutamate receptors 1 and 2 (GLUR1, GLUR2) and GFAP (Fig. 2E and Supplementary Fig. 6A), since GLUR2 is expressed in astrocytes, but not in Bergmann glia cells (Keinanen *et al.*, 1990; Iino *et al.*, 2001; Droste *et al.*, 2017). Colocalization of GLUR2 and GFAP antibodies indicates that most of the ectopic glial cells were astrocytes rather than transformed Bergmann glia cells. We performed additionally immunostaining against glial proteins BLBP and S100β that distinguishes radial glia—expressing BLBP and S100β—and astrocytes—expressing S100β alone (Supplementary Fig. 6B) and provides additional evidence for the invasion of reactive astrocytes into the molecular layer. Invasion of reactive astrocytes is a typical repair mechanism of the central nervous system (Sofroniew, 2005; Anderson *et al.*, 2014).

### P290R expression alters the morphology and number of Purkinje neuron synapses in the cerebellum

Purkinje neurons form glutamatergic synapses with parallel fibres or climbing fibres (CFs), and Bergmann glia cells not only provide a scaffold for the outgrowth of Purkinje neuron dendritic trees but also ensheath newly developing synapses (Palay and Chan-Palay, 1974; Grosche *et al.*, 1999; Yamada *et al.*, 2000; Lordkipanidze and Dunaevsky, 2005). Ultrastructural analyses illustrate that most of these synapses are almost completely surrounded by Bergmann glia cell processes in WT animals. This close wrapping of Purkinje neuron synapses by Bergmann glia cells could not be observed in the molecular layer of *Slc1a3^P290R/+^* animals (Supplementary Fig. 7).

**Figure 3:**
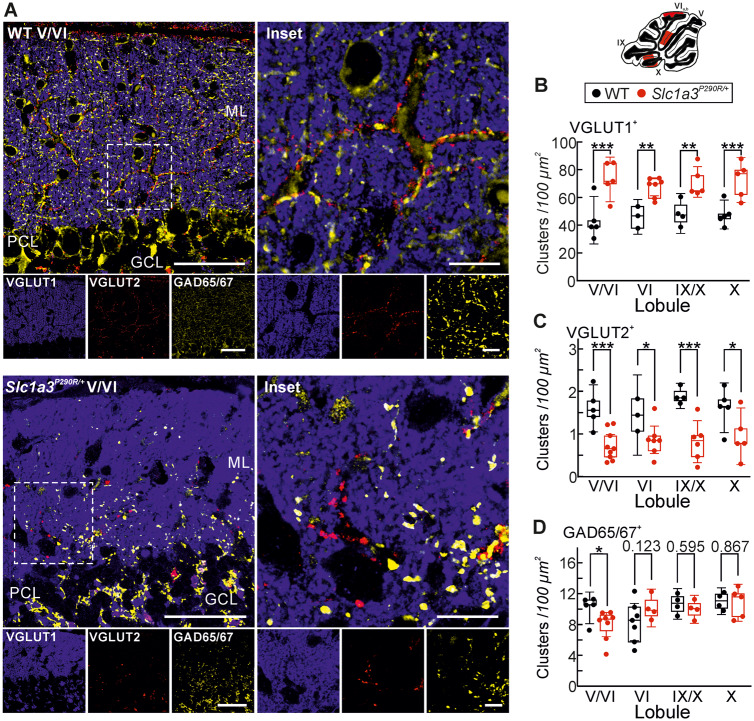
**Altered synapse densities in *Slc1a3^P290R/+^* cerebella.** Representative confocal images of WT and *Slc1a3^P290R/+^* cerebellar MLs (**A**) immunostained for the vesicular glutamate transporters VGLUT1 (blue, parallel fibres) and VGLUT2 (red, CFs) or for glutamate decarboxylase isoforms 65 and 67 (GAD65/67, yellow). Image **A** shows overviews and magnified insets. Median densities of glutamatergic VGLUT1^positive^ (**B**) and glutamatergic VGLUT2^positive^ (**C**) clusters of synaptic boutons are different in WT and mutant animals, whereas the densities of GABAergic clusters (**D**) are similar [*n *=* *3–5/5–7 in **B**; *n *=* *3–5/5–9 animals in **C**; *n *=* *4–7/4–8 animals in **D** (WT/Mut)]. Scale bars (**A**): overviews, 50 µm; insets, 10 µm. Data in **B**–**D** are shown as whisker-box plots with medians (whisker: ±CI, box: 25th to 75th percentiles with each data point representing the mean density from an individual animal; two-way ANOVA and Holm–Sidak *post hoc* test); *P ≤* *0.05, **P ≤* *0.01 and ***P ≤* *0.001; all *P*-values for this figure are provided in Supplementary Table 3. GCL, granule cell layer; ML, molecular layer; PCL, Purkinje cell layer.

During development the number of CFs in contact with a certain Purkinje neuron is reduced to one, and these changes are associated with the formation of parallel fibre synapses (Woodward *et al.*, 1974; Crepel, 1982; Mariani, 1982). To test for developmental changes in mutant animals, we quantified glutamatergic and GABAergic synapses in mutant animals by immunostaining parallel fibre synapses with anti-VGLUT1, CF synapses with anti-VGLUT2 (Miyazaki *et al.*, 2003) or anti-GAD65/67 antibodies, which label GABAergic synapses (Fig. 3 and Supplementary Material) in animals between P30 and P65. All tested cerebellar regions of mutant animals had an increased density of VGLUT1-positive clusters of presynaptic boutons (Fig. 3A and B), while VGLUT2-positive clusters are reduced in all tested cerebellar regions of mutant animals (Fig. 3A and C). The density of GAD65/67-positive synapses was slightly reduced in the region between lobules V and VI of mutant animals, whereas nodular regions had similar GAD65/67-positive cluster densities in both WT and mutant animals (Fig. 3A and D). Taken together, these findings demonstrate that expression of P290R modifies synaptic morphology and connections in the cerebellum.

### Increased P290R anion current is associated with glia cell apoptosis

**Figure 4:**
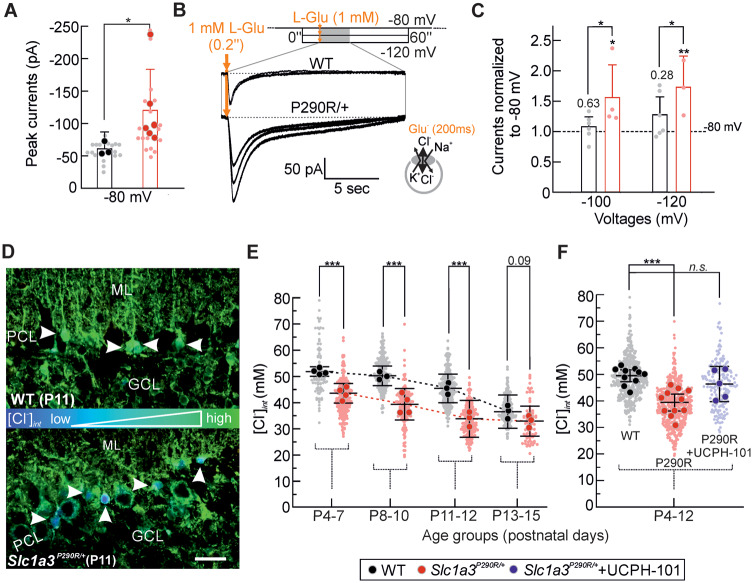
**Enhanced EAAT1/GLAST chloride currents in *Slc1a3^P290R/+^* Bergmann glia cells.** Bar graph **A** depicts pooled mean peak whole-cell current responses of Bergmann glia (−80 mV) to brief pulses (200 ms) of 1 mM l-Glu from experiments with acute cerebellar brain slices (±CI, *n *=* *3/6 animals, big circles, WT/Mut). Individual tested cells are shown as shaded small circles. In **B**, representative glutamate-induced Bergmann glia cell whole-cell currents are shown for three different holding potentials for WT and mutant cells. A bar graph **C** summarizes Bergmann glia peak current responses of individual WT and *Slc1a3^P290R/+^* cells for the voltages −100 and −120 mV, normalized to their respective currents at −80 mV (±CI, *n *=* *6/3–4 cells, WT/Mut). Representative FLIM recordings of cerebellar cortices (**D**) from WT and *Slc1a3^P290R/+^* mice at P11 reveal decreased [Cl^−^]_int_ in mutant Bergmann glia cells (arrows). [Cl^−^]_int_ is colour-coded according to the colour bar between images (scale bar: 25 µm). Point plots (**E**) depict mean [Cl^−^]_int_ during the first and second weeks of development for individual animals (big circles). [Cl^−^]_int_ of Bergmann glial cells was measured in WT and in *Slc1a3^P290R/+^* slices. Figure **F** summarizes data from the period with genotype-specific differences in the absence and in the presence of the EAAT1/GLAST-specific antagonist UCPH-101 (Abrahamsen *et al.*, 2013). In **A** and **C**, data points are means ± CI from individual animals (**A**, Mann–Whitney *U*-test) or cells (**C**, two-way ANOVA with repeated measures and Holm–Sidak *post hoc* tests). Data in **E** and **F** are presented and statistically analysed as means ± CI of individual animals [big circles, two-way ANOVA with Holm–Sidak *post hoc* test (**E**); Kruskal–Wallis ANOVA on ranks with Dunn’s *post hoc* test vs WT control (**F**)]. Distribution of measured individual cells is depicted in the background (**A**, **E** and **F**, small circles); *P ≤* *0.05, **P ≤* *0.01 and ***P ≤* *0.001; all *P*-values for this figure are provided in Supplementary Table 3. GCL, granule cell layer; ML, molecular layer; PCL, Purkinje cell layer.

P290R enhances EAAT1 anion currents in heterologous expression systems (Winter *et al.*, 2012), so that *Slc1a3^P290R/+^* Bergmann glia cells are expected to exhibit increased EAAT1/GLAST anion currents. We measured glutamate-elicited Cl^−^ currents using whole-cell patch clamping of Bergmann glia cells from acute brain slices of mice between P9 and P14 by applying brief pulses (200 ms) of glutamate (1 mM) at a holding potential near to their reversal potential (−80 mV). In these experiments, the pipette solution contained 145 mM KCl, 1 mM MgCl_2_ and 10 mM HEPES/KOH (pH 7.2). Bergmann glial cells were visually identified and distinguished from multipolar astrocytes—that are present in *Slc1a3^P290R/+^* molecular layers (Fig. 2E and G and Supplementary Fig. 6)—based on their size and location in proximity to Purkinje neurons. In a few cases, we inadvertently established the whole-cell configuration with neuronal cells, which could be easily distinguished from glial cells by their firing activity and discarded. Glutamate application elicited inward currents that were significantly increased in Bergmann glia cells from *Slc1a3^P290R/+^* mice (Fig. 4A, 60.7 ± 26.2/120.3 ± 63.2 pA, ±CI, *n *=* *3/6 animals, WT/Mut, *P *=* *0.024). Since P290R modifies the voltage dependence of EAAT1 anion currents and causes prominent activation by hyperpolarization (Winter *et al.*, 2012), we also compared glutamate-elicited currents of WT and *Slc1a3^P290R/+^* Bergmann glia cells at −100 and −120 mV with their respective currents at −80 mV. Hyperpolarization caused only minor current increases in WT Bergmann glia cells but significantly enhanced glutamate-elicited currents in *Slc1a3^P290R/+^* Bergmann glia cells (Fig. 4B and C).

Since EAAT1/GLAST anion channels are major determinants of the internal Cl^−^ concentration ([Cl^**−**^]_int_) in Bergmann glia cells (Untiet *et al.*, 2017), it was mandatory to test how increased EAAT1/GLAST anion currents affect [Cl^**−**^]_int_ in *Slc1a3^P290R/+^* Bergmann glia cells. We performed fluorescence lifetime imaging microscopy using the Cl^−^-sensitive dye MQAE in acute cerebellar slices (Untiet *et al.*, 2017) between P4 and P14 (Materials and methods, Fig. 4D–F). [Cl^**−**^]_int_ was lower in *Slc1a3^P290R/+^* mice than in WT, and incubation of *Slc1a3^P290R/+^* slices with the EAAT1/GLAST blocker 2-amino-5,6,7,8-tetrahydro-4-(4-methoxyphenyl)-7-(naphthalen-1-yl)-5-oxo-4*H*-chromene-3-carbonitrile (10 µM) increased the [Cl^**−**^]_int_ to values close to those of WT (Fig. 4F). In Bergmann glia cells, [Cl^**−**^]_int_ is in a dynamic equilibrium between Cl^**−**^ accumulation via NKCC transporters and Cl^**−**^ efflux through EAAT anion channels (Untiet *et al.*, 2017). Changes in numbers or transport rates of each protein shift the equilibrium and modify [Cl^**−**^]_int_. Reduced [Cl^**−**^]_int_ thus provides additional evidence for increased Cl^**−**^ efflux in *Slc1a3^P290R/+^* Bergmann glia cells.

We observed small difference in [Cl^**−**^]_int_ between WT and mutant mice also in the first postnatal week, i.e. before onset of glutamatergic innervation. EAAT1/GLAST is expressed at these ages (Schreiner *et al.*, 2014), and EAAT anion channels are not exclusively activated by external glutamate but also assume a basal activity under glutamate-free conditions or with internal glutamate (Fahlke *et al.*, 2016). Increased activity of P290R EAAT1/GLAST anion channels thus fully accounts for altered [Cl^**−**^]_int_ also at ages below P7.

**Figure 5:**
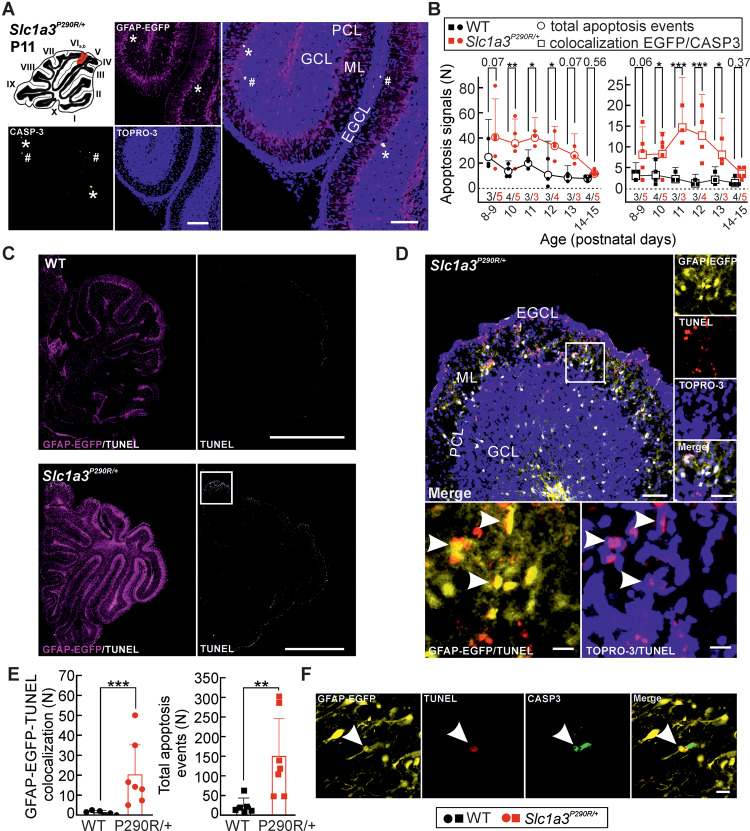
***Slc1a3^P290R/+^* Bergmann glia cell loss is caused by apoptosis during the second postnatal week.** Apoptosis of Bergmann glia cells visualized by confocal imaging (**A**) of an *Slc1a3^P290R/+^* cerebellar cortex immunostained for GFAP and CASP3 (scale bars: 50 µm) with colocalization of CASP3/GFAP–EGFP (asterisks) and CASP3 (hash) signals in a transgenic mouse. Point plots of mean numbers of total CASP3^positive^ cells and CASP3^positive^ Bergmann glia of individual animals over time (**B**) demonstrate increased numbers of apoptotic Bergmann glia cells in *Slc1a3^P290R/+^* animals during the second postnatal week. Confocal images from whole sagittal cerebellar slices stained for fragmented DNA (**C**, EGFP/TUNEL) and magnified views from region of interest (**D**) of mutant cerebellum as indicated in **C**. Merged views of EGFP/TUNEL and of TOPRO-3/TUNEL show the overlap of GFAP–EGFP and TUNEL signals, and the localization of TUNEL signals in the nuclei (**C**, arrowheads). Bar graphs in **B** show the mean numbers (±CI) of apoptotic signals (left) and the mean numbers of EGFP/CASP3 colocalized signals (right) per individual animal (*n *=* *3–5 animals per genotype and age group, two-way ANOVA with Holm–Sidak *post hoc* test). Bar graphs in **E** show the mean total numbers of TUNEL signals for individual animals (*n *=* *6/7, WT/Mut, left) and the mean numbers (±CI) of GFAP–EGFP/TUNEL colocalized signals for the same animals (right). Statistical analyses were done with Kruskal–Wallis ANOVA on ranks and Dunn’s *post hoc* test for colocalized signals (left) and total apoptosis events (right), separately. Confocal image (**F**) shows the colocalization of EGFP/TUNEL/CASP3. Scale bars: 100 µm (**A**), 1 mm (**C**); 100, 25 and 10 µm (**D**); and 10 µm (**F**); *P ≤* *0.05, **P ≤* *0.01 and ***P ≤* *0.001; all *P-*values for this figure are provided in Supplementary Table 3. EGCL, external granule cell layer, GCL, granule cell layer, ML, molecular layer, PCL, Purkinje cell layer.

[Cl^**−**^]_int_ was smaller in *Slc1a3^P290R/+^* than in WT Bergmann glia cells but still larger than expected for passive distribution. Thus, activation of EAAT1/GLAST and EAAT2/GLT-1 anion channels at the onset of glutamatergic synaptic transmission during the second postnatal week (Watanabe and Kano, 2011) results in Cl^**−**^ efflux from Bergmann glia cells in both *Slc1a3^P290R/+^* and WT animals. These currents are larger in *Slc1a3^P290R/+^* as in WT animals and might cause cell shrinking and apoptosis of mutant Bergmann glia cells (Porcelli *et al.*, 2004; Ernest *et al.*, 2008). We crossed reporter mice with GFAP–EGFP with *Slc1a3^P290R/+^* animals, stained cerebellar tissue from P9–P15 with antibodies for active caspase**-**3 (CASP3) and identified apoptotic glial cells by the colocalization of EGFP and CASP3 (Fig. 5A and B). Figure 5B shows the numbers of apoptotic cells from WT and *Slc1a3^P290R/+^* brain slices during postnatal development, demonstrating significantly increased levels of apoptotic events in mutant cerebella (Fig. 5B, left) and significantly more apoptotic Bergmann glial cells (Fig. 5B, right) between P10 and P13 in the animal model. Since CASP3 activation was also reported during normal development (Oomman *et al.*, 2005), we additionally tested the apoptosis marker TUNEL in GFAP–EGFP-expressing mice of both genotypes (Gavrieli *et al.*, 1992). We observed substantially more TUNEL signals in mutant than in WT cerebella (Fig. 5C). In mutant animals, TUNEL colocalizes with the nuclei of cells expressing GFAP–EGFP in the Purkinje cell layer, indicating glia apoptosis (Fig. 5D and E). The number of apoptotic Bergmann glia cells detected by TUNEL/GFAP–EGFP colocalization was comparable to the CASP3 assay between P10 and P13 (CASP3: 10.2 ± 2.9, TUNEL: 20.2 ± 15.1, means ± CI, *n *=* *15/7 mutant animals, CASP3/TUNEL). We also observed increased numbers of TUNEL and of CASP3 signals that did not colocalize with GFAP (Fig. 5A and B–E, CASP3: 33.6 ± 6.2, TUNEL: 149.4 ± 96.8, means ± CI*, n *=* *15/7 mutant animals, CASP3/TUNEL). These signals might be due to earlier apoptosis events of Bergmann glial cells or due to apoptosis of other cell types, such as migrating granule cells in the external granule cell layer.

### Abnormal spiking properties of *Slc1a3^P290R/+^* Purkinje neurons

**Figure 6:**
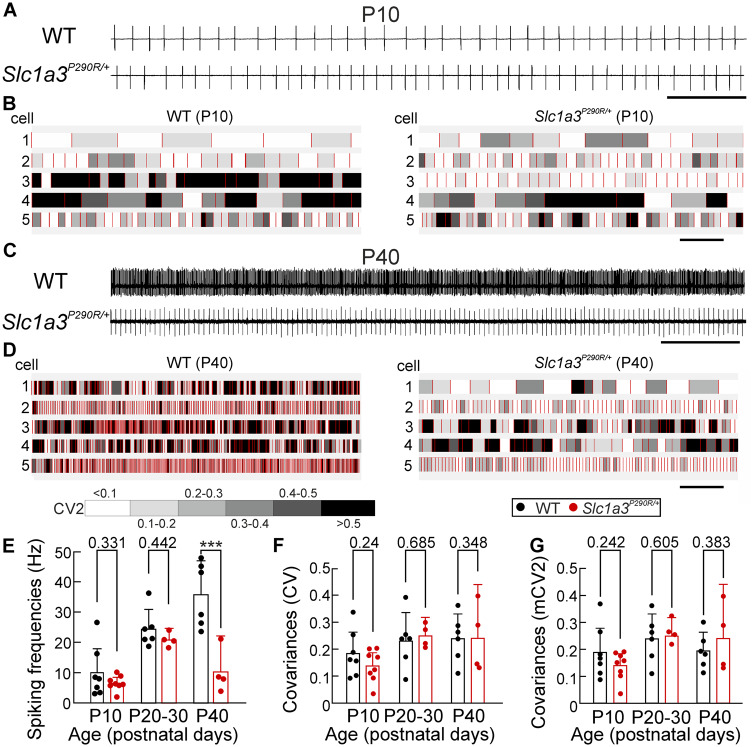
***Slc1a3^P290R/+^* Purkinje neurons display abnormal spiking properties.** Current traces obtained from cell-attached patch clamp recordings (**A** and **C**) show patterns of spontaneous firing from individual Purkinje neurons in acute slices from juvenile (**A**, P10) or adult (**C**, P40) WT or *Slc1a3^P290R/+^* mice. Burst plots (**B** and **D**) of representative Purkinje neuron spike trains for juvenile (**B**) or adult (**D**) WT or *Slc1a3^P290R/+^* mice. Spikes are shown as red bars and interspike intervals in grey scale according to the temporary covariance (CV2). Purkinje neuron mean simple spike frequencies (**E**) differ between P40 WT and mutant animals, whereas mean absolute CVs (**F**) and mean CVs of interspike intervals (**G**) are similar in WT and mutant animals. Pooled data are shown as bars with means ± CI, with **E**–**G** representing mean values from the same animals (P10, *n *=* *7/8; P20–30, *n *=* *6/4; P40, *n *=* *6/4 animals, WT/Mut, two-way ANOVA with Holm–Sidak *post hoc* tests; ***P ≤* *0.001); all *P*-values for this figure are provided in Supplementary Table 3.

Cerebellar Purkinje neurons represent the only neuronal output of the cerebellar cortex (Marr, 1969). In the absence of synaptic input, they fire action potentials with precisely regulated interspike intervals (Bell and Grimm, 1969; Arancillo *et al.*, 2015) and changes in the frequency and in the temporal precision of interspike intervals are known to impair motor coordination (Hoebeek *et al.*, 2005; Walter *et al.*, 2006; Alvina and Khodakhah, 2010; Jayabal *et al.*, 2017). We measured spontaneous simple spiking activity with cell-attached patch clamp recordings (Donato *et al.*, 2006) in vermal regions in lobes V/VI, VI–VIII and VIII/IX of acute WT and mutant cerebellar slices. Representative recordings from WT and *Slc1a3^P290R/+^* mice at P10 (Fig. 6A) and P40 (Fig. 6C) demonstrate typical biphasic spikes, corresponding to inward and outward currents during action potentials (Womack and Khodakhah, 2002). In Purkinje neurons from WT animals, the spontaneous spiking frequency increases during development from 10.1 ± 7.9 Hz at P10 to 35.8 ± 11.8 Hz at P40 (±CI*, n *=* *7/6 animals). In *Slc1a3^P290R/+^* animals, the developmental acceleration was less pronounced, resulting in significantly slower firing in mutant Purkinje neurons at P40 (10.4 ± 11.9 Hz, ±CI, *n *=* *4 animals) (Fig. 6E).

To compare the temporal precision of simple spiking activities, we calculated ISI covariances (CV) and intrinsic variabilities (CV2) for WT and mutant Purkinje neurons (Holt *et al.*, 1996). For all tested ages, CV and CV2 were not different between WT and mutant Purkinje neurons (Fig. 6F and G). The CV values observed in *Slc1a3^P290R/+^* animals were in good agreement with other reports studying acute slices at similar temperatures (Wulff *et al.*, 2009; Jayabal *et al.*, 2017). Smaller CV values (<0.1) were only reported in acute slices from older mice (Jayabal *et al.*, 2016) or in studies at physiological temperatures (Hansen *et al.*, 2013). We conclude that the developmental acceleration of simple spike frequencies (McKay and Turner, 2005) is absent in *Slc1a3^P290R/+^*, probably due to progressive glia and neuron degeneration.

### Loss of Bergmann glia modifies climbing fibre regulation of Purkinje neuron activity in *Slc1a3^P290R/+^* mice

Bergmann glial processes ensheath glutamatergic synapses between CFs and Purkinje neurons (Supplementary Fig. 7), and glutamate uptake into Bergmann glial processes helps reducing glutamate spill-over from the synaptic cleft after the simultaneous release of multiple synaptic vesicles (Wadiche and Jahr, 2001). The CF–Purkinje neuron synapse thus represents a system well suited to study the functional consequences of EAAT1/GLAST dysfunction and Bergmann glia degeneration on glutamatergic synaptic transmission in the cerebellum.

**Figure 7:**
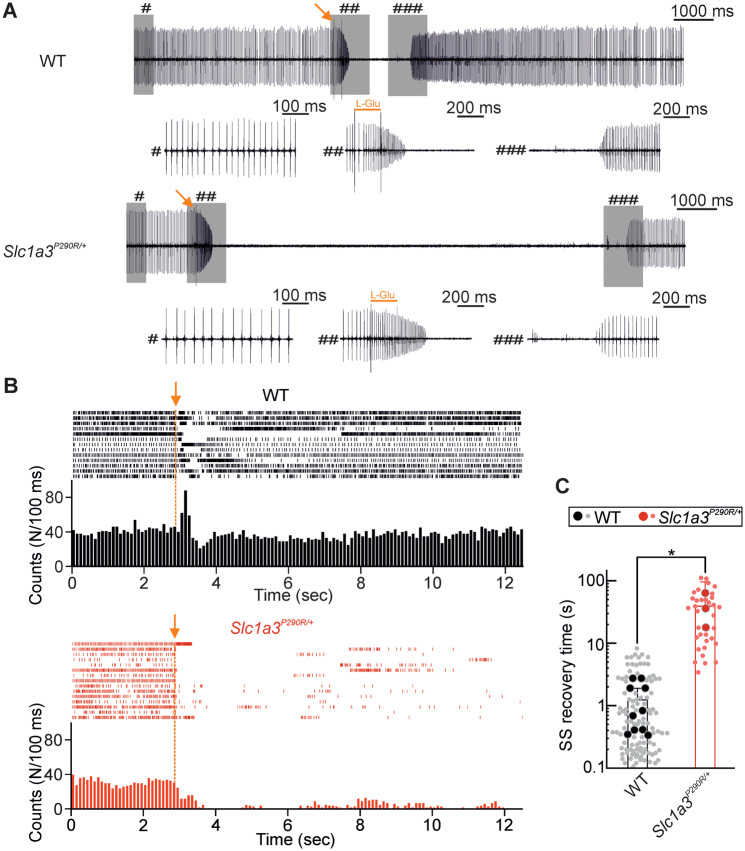
**Increased CF pause durations in *Slc1a3^P290/+^* Purkinje neurons.** Current traces from cell-attached patch clamp recordings of Purkinje neuron somata (**A**) show representative simple spike activities of WT and *Slc1a3^P290R/+^* Purkinje neurons before and after glutamate puff application (arrow). Pooled burst plots with corresponding histograms (**B**) from individual responses of WT and *Slc1a3^P290R/+^* Purkinje neurons show substantial prolonged recovery periods (**C**) for Purkinje neurons from *Slc1a3^P290R/+^* mice. Burst plots (**B**) are constructed from glutamate puff responses of *n *=* *7/3 Purkinje neurons (WT/Mut), and histograms are binned at 100 ms. Bar plot (**C**) shows recovery times as means ± CI from mean pause lengths values observed in *n *=* *10/3 individual animals (WT/Mut, big circles, Mann–Whitney *U*-test) and pooled glutamate responses from all tested cells (small circles). Lengths of scale bars are indicated in **A**; *P ≤* *0.05; *P*-value for this figure is provided in Supplementary Table 3.

CF activity typically interrupts simple spike activity for periods up to several hundred milliseconds, the so-called CF pauses (Eccles *et al.*, 1966; Bell and Grimm, 1969; Latham and Paul, 1971; Sato *et al.*, 1992). These pauses play a role in signal transmission to the deep cerebellar nuclei during learning processes (Otis *et al.*, 2012). We measured the lengths of these pauses in WT and *Slc1a3^P290R/+^* Purkinje neurons after pulses with supersaturating glutamate concentrations at ages below P30. At these ages, simple spike activity was similar for WT and mutant Purkinje neurons (Fig. 6E). Figure 7A depicts representative responses from WT and mutant Purkinje neurons to glutamate application, and in Fig. 7B, aligned simple spike events were plotted vs time with corresponding histograms of aligned events binned at 100 ms, before and after the glutamate puff. In WT Purkinje neurons, the glutamate application evokes a short period of augmented spike firing, followed by a short depressed phase as described earlier (Tang *et al.*, 2017). While the baseline simple spike activities are recovered within several seconds after the pulse in WT Purkinje neurons (1.2 ± 0.7 s) in most cases, silent periods were significantly longer in *Slc1a3^P290R/+^* Purkinje neurons (39.2 ± 57.2 s, ±CI, *n *=* *10/3 animals, *P *=* *0.011, Fig. 7C). We conclude that glutamate uptake is severely reduced in *Slc1a3^P290R/+^* mice and results in altered synaptic transmission between CFs and Purkinje neurons.

### Cerebellar degeneration in *Slc1a3^P290R/+^* mice

**Figure 8:**
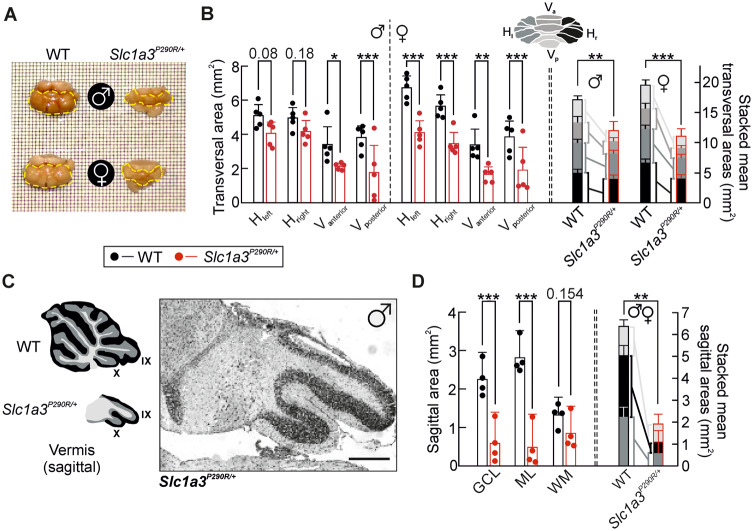
**Cerebellar degeneration in old *Slc1a3^P290R/+^* mice.** Overall views of fixed brains (**A**) from adult WT or *Slc1a3^P290R/+^* mice (>P180) of both sexes on a grid surface (grid size: 0.25 mm^2^) show degeneration of cerebella from transgenic animals. Bar graphs (**B**) show details of size decreases by comparing different cerebellar areas for male and female animals, and stacked bars with summed areas of WT and *Slc1a3^P290R/+^* cerebella show overall cerebellar size decreases for both sexes. Sagittal views of Nissl-stained vermis region (**C**) from a male 2.5-year-old (P921) *Slc1a3^P290R/+^* mouse. Insets demonstrate the orientation of the slice and the vermis layer structures for WT and *Slc1a3^P290R/+^* mice. Quantification of mean sagittal size distributions (**D**) of vermal cerebellar layers in elderly WT and mutant mice (∼P600–P950) of both sexes shows that ML and granule cell layer denote substantial mass decreases, but not WM, and stacked bars with summed areas of WT and *Slc1a3^P290R/+^* cerebella show overall sagittal cerebellar size decreases. Bar graphs in **B** and **D** denote mean area sizes ±CI and in stacked bars; sizes are presented as means ± SEM. Each mean was calculated from *n *=* *5^♂^5^♀^/5^♂^5^♀^ (**B**) and *n *=* *4/4 (**D**) animals (WT/Mut). Data of both sexes were separately analysed with two-way ANOVA and Holm–Sidak *post hoc* tests. Colour coding of cerebellar areas/layers is indicated in the insets in **C** and **D**. Scale bar: 250 µm (**C**); *P ≤* *0.05, **P ≤* *0.01 and ***P ≤* *0.001; all *P*-values for this figure are provided in Supplementary Table 3. H, hemisphere; V, vermis; GCL, granule cell layer; ML, molecular layer; WM, white matter.

We reasoned that the absence of Bergmann glia cells might result in cerebellar atrophy and compared the sizes of WT and *Slc1a3^P290R/+^* cerebella in old mice (i.e. >P180). Quantification of cerebellar transversal areas revealed smaller values in all tested *Slc1a3^P290R/+^* than in corresponding WT animals (Fig. 8A), due to a reduction in all cerebellar regions (Fig. 8B). We observed similar alterations in mutant female and male mice (*n *=* *5/5 animals, Mut^♂^/Mut^♀^, *P *=* *0.55) and thus pooled results from both sexes in subsequent analyses. The mean size was reduced by 37 ± 13% (±*σ*, *n *=* *10/10 animals, WT/Mut) in *Slc1a3^P290R/+^*, and 6 out of 10 cerebella were reduced by >35% in transversal sizes. Nissl staining of slices from whole vermis regions of mutant mice shows fewer cerebellar lobes and a smaller total sagittal plane, with smaller granule cell layers and molecular layer areas (Fig. 8C and D). There was no obvious size reduction in white matter area of *Slc1a3^P290R/+^* slices (*n *=* *4/4 animals, WT/Mut, Fig. 8D). We additionally compared the number of Purkinje cells and the thickness of molecular layers from younger WT and mutant animals (P27–60) from lobes VI and X. There was no difference in molecular layer thickness between genotypes (*P *=* *0.391). The number of Purkinje cells was significantly reduced in the external region of mutant lobes VI (4.4 ± 0.5/3.7 ± 1.3 Purkinje neurons/100 µm, ±CI, *n *=* *8/11, WT/Mut*, P = *0.003), but not in the other tested regions (Supplementary Fig. 8). We conclude that loss of Bergmann glia cells leads to generalized cerebellar degeneration in *Slc1a3^P290R/+^* mice, likely because Bergmann glial apoptosis impairs glutamate removal and causes excitotoxic Purkinje neuron death.

## Discussion

EA type 6 is an inherited condition characterized by impaired motor coordination with epilepsy and migraine-like headache. Thus far, all reported cases have been associated with mutations in *SLC1A3* (Jen *et al.*, 2005; de Vries *et al.*, 2009; Pyle *et al.*, 2015; Choi *et al.*, 2017a, *b*; Iwama *et al.*, 2018), but how these mutations cause the specific clinical phenotype of EA is still insufficiently understood. To answer this question, we used a knock-in mouse model expressing EAAT1/GLAST carrying a mutation recently described in a human patient (Jen *et al.*, 2005). *Slc1a3^P290R/+^* mice suffer from epileptic attacks, ataxia and cerebellar atrophy and thus exhibit similar neurological symptoms as the patient with the same mutation. Disease-associated *SLC1A3* mutations were initially assumed to modify glutamate transport rates and to affect synaptic transmission via decreased glutamate uptake. However, comparison of the neurological symptoms of *Slc1a3^P290R/+^* and the corresponding knock-out animal *Slc1a3*^−/−^—that lacks completely EAAT1/GLAST-mediated glutamate uptake and only suffers from moderate impairment of motor coordination (Watase *et al.*, 1998; Perkins *et al.*, 2016)—argues against this assumption. We here demonstrate that the expression of P290R EAAT1 reduces glutamate uptake by a different mechanism: gain-of-function of EAAT1/GLAST Cl^−^ currents significantly impairs cerebellar glutamate homeostasis by causing Bergmann glia degeneration.

P290R causes gain-of-function of *h*EAAT1 anion channel activity in heterologous expression systems (Winter *et al.*, 2012), and we found significantly increased glutamate-induced anion currents in *Slc1a3^P290R/+^* Bergmann glia cells. Although Bergmann glia [Cl^**−**^]_int_ was slightly lower in mutant than in WT, these values were significantly larger than expected for passive distributions (Fig. 4). Increased P290R EAAT1/GLAST anion channel activity will thus result in enhanced Cl^**−**^ efflux and excessive cell shrinkage in *Slc1a3^P290R/+^* Bergmann glia cells upon establishment of glutamatergic synaptic signalling in the second postnatal week. Cl^**−**^ loss and cell shrinkage can trigger apoptosis (Walev *et al.*, 1995; Friis *et al.*, 2005), and EAAT1/GLAST anion channel gain-of-function can thus account for glial apoptosis in *Slc1a3^P290R/+^* at ages between P9 and P14 (Figs 4 and 5). We were not able to show cell shrinkage in mutant animals directly, most likely since cell shrinkage leads to apoptosis in a fast and irreversible manner. EAAT1/GLAST is highly expressed in glial cells in the cerebellum, so Bergmann glia cell apoptosis is likely to be the first stage of cerebellar degeneration (Figs 2 and 5).

We observed Bergmann glial apoptosis with two markers, TUNEL and CASP3 (Fig. 5). Recent reports (Oomman *et al.*, 2004; Oomman *et al.*, 2005) described a constitutive expression of CASP3 in a large percentage of rat Bergmann glial cells, whereas other apoptosis markers as TUNEL or Annexin V were absent. We could only detect CASP3 expression in apoptotic cells in mutant animals, but not in WT Bergmann glial cells, suggesting species-specific roles of CASP3 in mouse and rat.

Spontaneous spiking of Purkinje neurons is controlled by feed-forward inhibition by interneurons (Marr, 1969; Midtgaard, 1992; Hausser and Clark, 1997; Wulff *et al.*, 2009) and thus represents a marker for the integrity of the cerebellar network. Although Purkinje neuron activity was comparable in young WT and mutant animals, degenerative processes result in lower frequency spiking in adult *Slc1a3^P290R/+^* mice (Fig. 6). Similar changes in Purkinje neuron activity were observed in another ataxia animal model, the ataxin 2 (*Atxn2*) mouse carrying a mutation of ataxin 2 [ATXN2^Q127^: Tg(Pcp2-ATXN2*127Q)#Plt/0] found in patients with spinocerebellar ataxia type 2. These animals exhibited cerebellar degeneration and decreased Purkinje neuron firing rates, with no major difference in firing precision (Hansen *et al.*, 2013).

Purkinje neurons receive glutamatergic inputs from olivary CFs, with synaptic activity usually followed by a period without spontaneous spiking (Eccles *et al.*, 1966; Bell and Grimm, 1969; Latham and Paul, 1971; Sato *et al.*, 1992), the so-called CF pause. The prominent Bergmann glia degeneration in *Slc1a3^P290R/+^* causes a significant reduction in glutamate uptake in these animals that results in increased CF pause durations in Purkinje neurons of these mice (Fig. 7). Changes in CF pause durations are a common finding in mouse models for ataxia and hemiplegic migraine, for example in a migraine mouse model carrying a mutation in *Cacna1a* (Gao *et al.*, 2012) or in ataxic mice lacking BK channels (Cheron *et al.*, 2009).

*Slc1a3^P290R/+^* animals exhibit the degradation of Bergmann glia in the cerebellum, but not of glial cells in other brain regions such as hippocampus or cortex. This difference is likely due to separate expression levels of EAAT1/GLAST and/or distinct [Cl^**−**^]_int_ in Bergmann glia. Insertion of the homologous P > R mutation into *Drosophila* EAAT1 (Eaat1^P>R^) or expression of mutant human EAAT1 caused episodic paralyzes with compromised astrocyte morphology and developmental impairment in *Drosophila* larvae (Parinejad *et al.*, 2016). This phenotype was mimicked by the overexpression of K^+^-Cl^**−**^ cotransporter and could be rescued by Na^+^-K^+^-2Cl^**−**^ cotransporter expression. These findings highlight the physiological importance of [Cl^−^]_int_ homeostasis for glial function but also identify differences between mouse Bergmann glia cells and *Drosophila* glial cells: Bergmann glial apoptosis is an initial stage of cerebellar degeneration in *Slc1a3^P290R/+^* mice, while Eaat1^P>R^ larvae have impaired astrocyte infiltration but no degeneration. Glial cells express various Cl^−^-coupled transporters, whose function might be modified by reduced [Cl^−^]_int_ in *Slc1a3^P290R/+^* mice. Glial GATs are Na^+^/Cl^−^/GABA transporters, and the observed changes in [Cl^−^]_int_ would cause a reduction in resting [GABA] by ∼20% between P10 and P12. Cl^−^/HCO_3_^−^ exchange might modify intracellular pH in *Slc1a3^P290R/+^*glia. However, since the main alteration in *Slc1a3^P290R/+^* is a dramatic reduction in the number of Bergmann glia cells, all these predicted changes are minor compared with the morphological changes.

So far, five different brain disorders have been associated with *SLC1A3* mutations: EA (Jen *et al.*, 2005; de Vries *et al.*, 2009; Choi *et al.*, 2017*a*; Iwama *et al.*, 2018), migraine (Kovermann *et al.*, 2017); Tourette syndrome (Adamczyk *et al.*, 2011), attention-deficit hyperactivity disorder and autism (van Amen-Hellebrekers *et al.*, 2016). Although the underlying physiological mechanisms are insufficiently understood, these conditions might share fundamental pathophysiological processes: a sequence variant predicting E219D, found in some individuals with Tourette syndrome (Adamczyk *et al.*, 2011), increases the relative probability of surface membrane insertion for human EAAT1 and *SLC1A3* duplication is likely to increase EAAT1 expression in attention-deficit hyperactivity disorder and autism. In these diseases, increased EAAT1 numbers in the surface membrane are expected to enhance glutamate-activated anion currents in glial cells. It is tempting to speculate that these changes might alter glial function and neuronal migration, resulting in changes in neuronal network formation and function.

EAAT glutamate transporters are prototypical dual function proteins that mediate secondary-active glutamate transport and anion conductance. Although the structural basis of these two different transport processes has been established (Machtens *et al.*, 2015), the physiological impact of linking these distinct functions remains less clear. It is now clear that EAATs play a major role in regulating glial [Cl^**−**^]_int_ in the mammalian central nervous system (Untiet *et al.*, 2017) and in glial differentiation in *Drosophila* (Parinejad *et al.*, 2016). Our work illustrates how changes in EAAT1 anion channel activity can have dramatic consequences on differentiation and integrity of the cerebellum. It has been known for decades that glial and neuronal EAATs have differences in anion channel activity (Wadiche *et al.*, 1995). Our findings highlight the impact of low glial EAAT anion channel activity on glial Cl^**−**^ homeostasis. Animals carrying a heterozygous P290R mutation have only moderately increased EAAT-mediated anion currents but exhibit dramatic changes in cerebellar differentiation and profound cerebellar degeneration. Hence, glial EAATs must be optimized for both effective secondary-active transport and low anion channel activity.

## Supplementary Material

fcaa022_Supplementary_Data

## Abbreviations

BLBP =: brain lipid-binding protein
CASP3 =: caspase 3
CF =: climbing fibre
CV =: covariances
[Cl^−^]_int_ =: internal chloride concentration
EAAT1 =: excitatory amino acid transporter 1
EAs =: episodic ataxias
EGFP =: enhanced green fluorescent protein
GFAP =: glial fibrillary acidic protein
GLAST =: glutamate–aspartate transporter
MQAE =: 1-(ethoxycarbonylmethyl)-6-methoxy-quinolinium bromide
TUNEL =: terminal dUTP nick-end labelling
WT =: wild type
