# Supplementary material for: Increased glutamate transporter-associated anion currents cause glial apoptosis in episodic ataxia 6
Source: Brain Commun. 2020 Mar 4;2(1):fcaa022. doi: 10.1093/braincomms/fcaa022 (PMC7425361; doi:10.1093/braincomms/fcaa022)
Supplement: fcaa022_Supplementary_Data [file fcaa022_supplementary_data.zip › Supplementary_Figures.pdf]

Supplementary Figures
for
Increased glutamate transporter-associated anion currents cause glial apoptosis in episodic ataxia 6

Peter Kovermann[1], Verena Untiet[3], Yulia Kolobkova[1], Miriam Engels[1],Stephan Baader[2], Karl Schilling[2], and Christoph Fahlke[1]

[1]Institut für Biologische Informationsprozesse, Molekular- und Zellphysiologie (IBI-1), Forschungszentrum Jülich, 52425 Jülich, Germany

[2]Anatomisches Institut, Rheinische Friedrich-Wilhelm-Universität, 53115 Bonn, Germany

[3]present address: Center for Translational Neuromedicine, Københavns Universitet, 2200 København N, Denmark

Table of content for Supplementary Figures:

Supplementary Fig. 1: Targeting strategy of *Slc1a3*$^{P290R/+}$ transgene insertion, page 2.

Supplementary Fig. 2: Fitness of WT and transgenic mice, page 3.

Supplementary Fig. 3: Spin failures case and error latencies on the rotarod, page 3.

Supplementary Fig. 4: Immunostaining of Bergmann glial cells and western blotting of cerebellar lysates with specific Bergmann glial markers, page 4

Supplementary Fig. 5: Semisections from the cerebella at P50 page 5.

Supplementary Fig. 6: Glial cell types in the molecular layer of *Slc1a3*$^{P290R/+}$, page 6.

Supplementary Fig. 7: Transmission electron microscopy, page 7.

Supplementary Fig. 8: Molecular layer thickness and Purkinje neuron numbers, page 8.

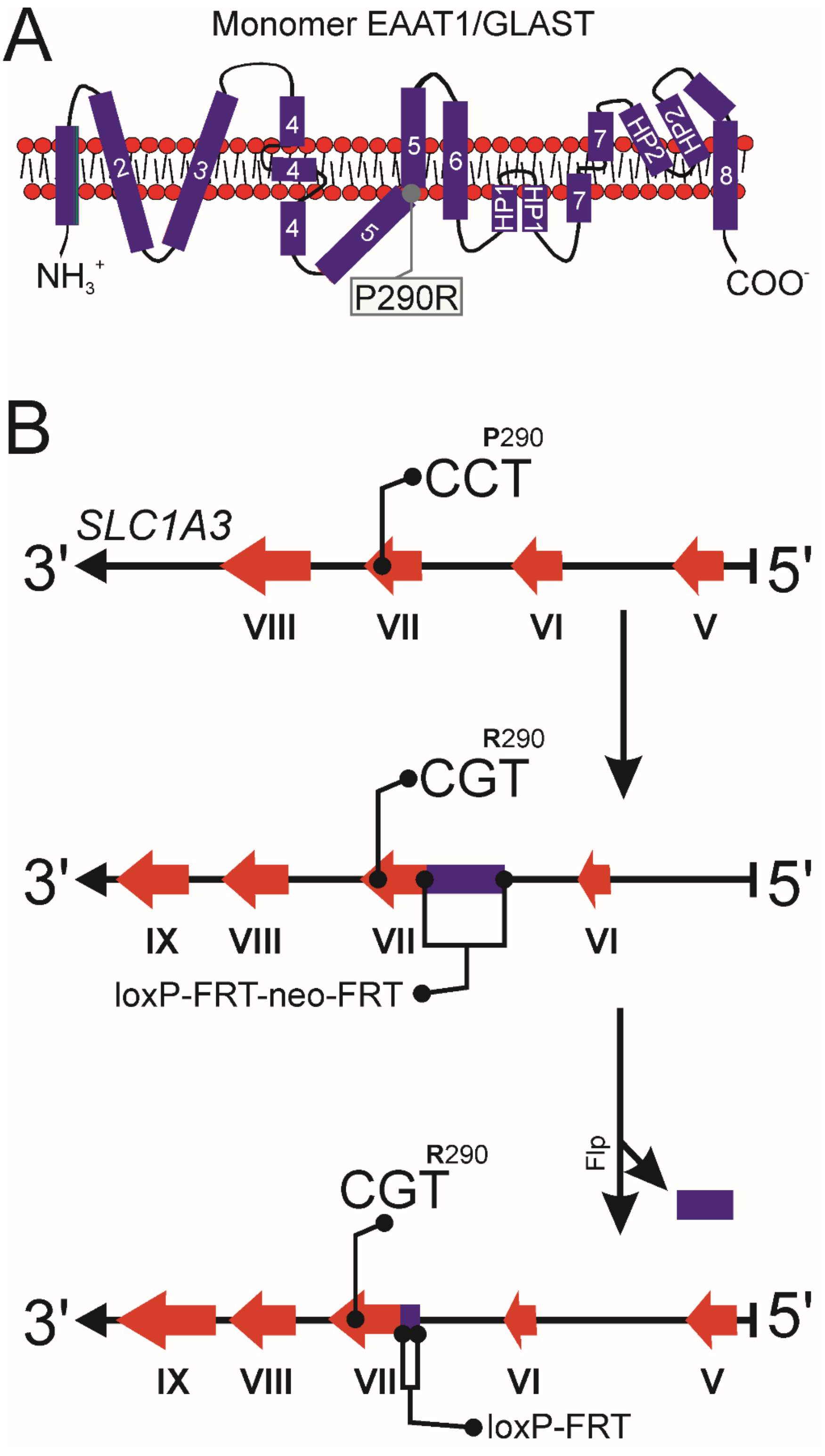

Supplementary Figure 1 – Targeting strategy of *Slc1a3*$^{P290R/+}$ transgene insertion. Transmembrane topology of EAAT1 monomers (**A**) and localization of the disease-associated mutation P290R. Schematic diagram (**B**) showing the location of the P290R mutation in the gene *Slc1a3* with target codons for the WT *Slc1a3*-targeting construct used for homologous recombination (B, top). Recombinase-mediated nucleotide exchange inserts the mutation and an FRT-flanked neomycin cassette (for selection) into exon VII (SA, short arm; LA, long arm; neo, neomycin, B, middle). The mutated *Slc1a3* gene after Flp-mediated excision of the neomycin cassette (B, bottom).

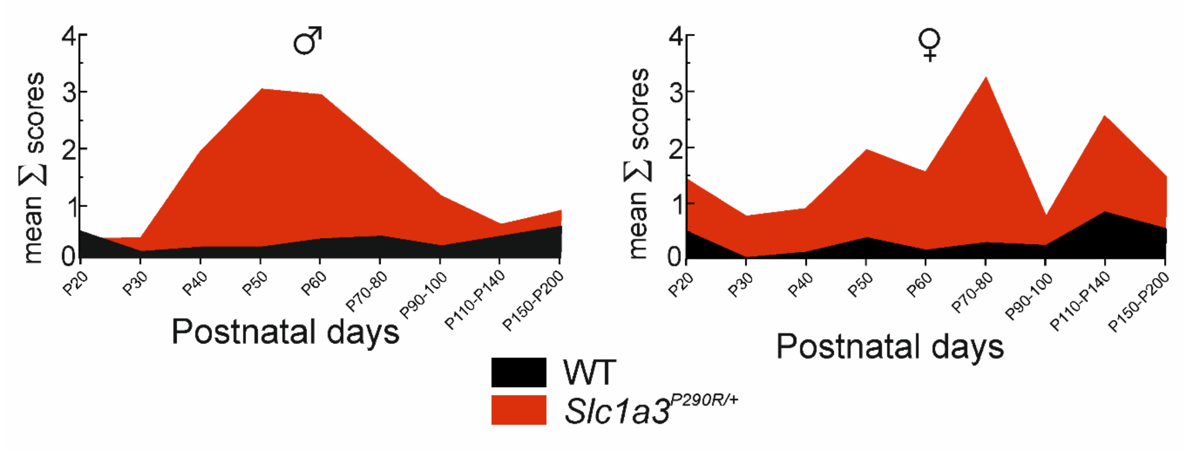

Supplementary Figure 2 – Fitness of WT and transgenic *Slc1a3*$^{P290R/+}$ mice. Age dependences of fitness for WT and *Slc1a3*$^{P290R/+}$ mice of mean sum of scores (see Supplementary Table 2) for male (left) and female (right) animals. These data represent the mean scores of 21/21 ♂ and 10/14 ♀ (WT/Mut) regularly scored animals.

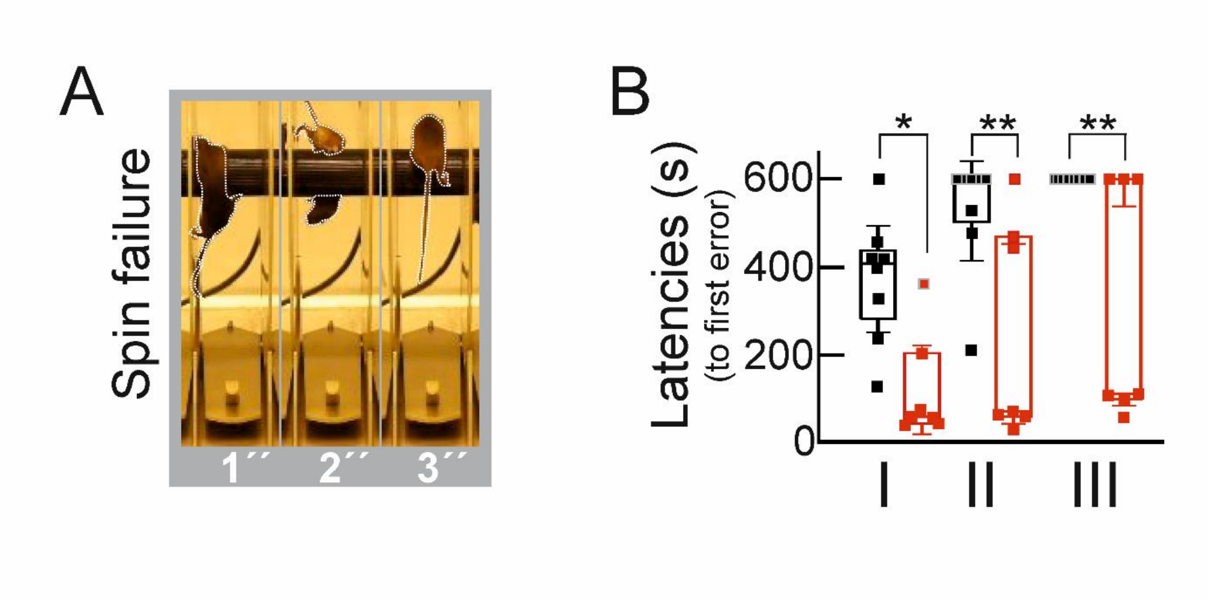

Supplementary Figure 3 – Spin failure case and error latencies of WT and mutant mice on the rotarod. The image series shows a representative case of a spin failure during the rotarod test, where a mouse is rotating with the waltz (**A**). Graph (**B**) depicts analysis of first error latencies for WT (black) and *Slc1a3*$^{P290R/+}$ (red) mice without differentiation between *spin*- and *fall*- errors for the rotarod test shown in Fig. 1 (2-*way* ANOVA with repeated measures and Holm-Sidak *post hoc* tests); all *P*–values are available online for this figure (Supplementary Table 3).

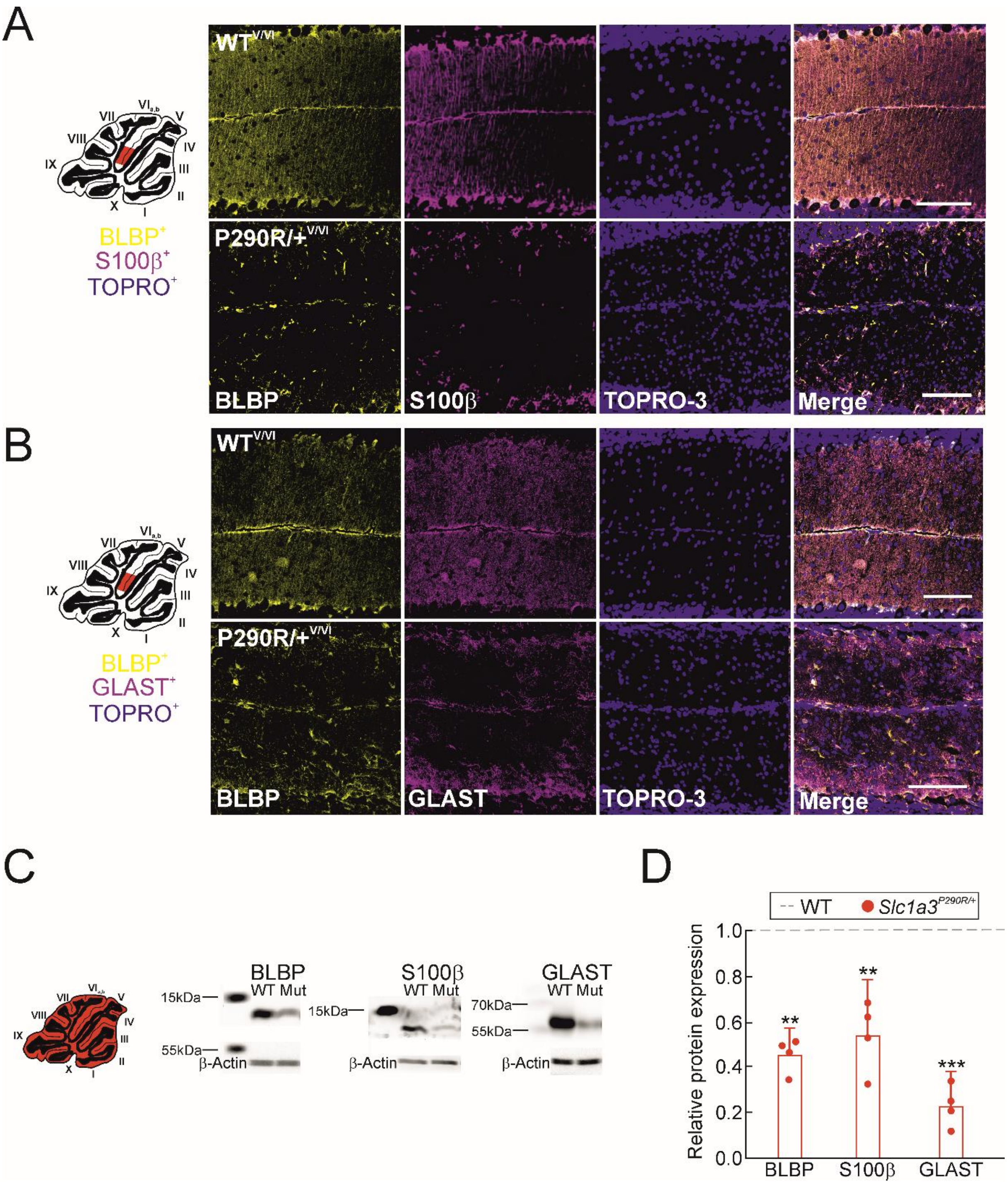

Supplementary Figure 4 – Immunostaining of Bergmann glial cells with specific markers. Confocal images of cerebella from WT and *Slc1a3*$^{P290R/+}$ mice immunostained against glial markers BLBP, S100β and BLBP, GLAST (**A**, **B**) show the differences in protein expression and cellular distribution between cerebella from WT and mutant mice. Western blots from whole cerebella lysates (**C**) depict relative protein expression of BLBP, S100β, and GLAST for WT and *Slc1a3*$^{P290R/+}$ cerebella. Bar graphs providing relative intensities of western blot signals (**D**) illustrate significant reduction of tested Bergmann glial markers a in *Slc1a3*$^{P290R/+}$. In D the relative expression of glial markers is shown as bars with means (±*CI*), after normalization to WT. Each marker was tested in cerebellar lysates from n = 3–4 / 4 (WT/Mut) different animals (2-*way* ANOVA and Holm-Sidak *post hoc* tests); all *P*–values are available online for this figure (Supplementary Table 3). Scale bars: 100 µm.

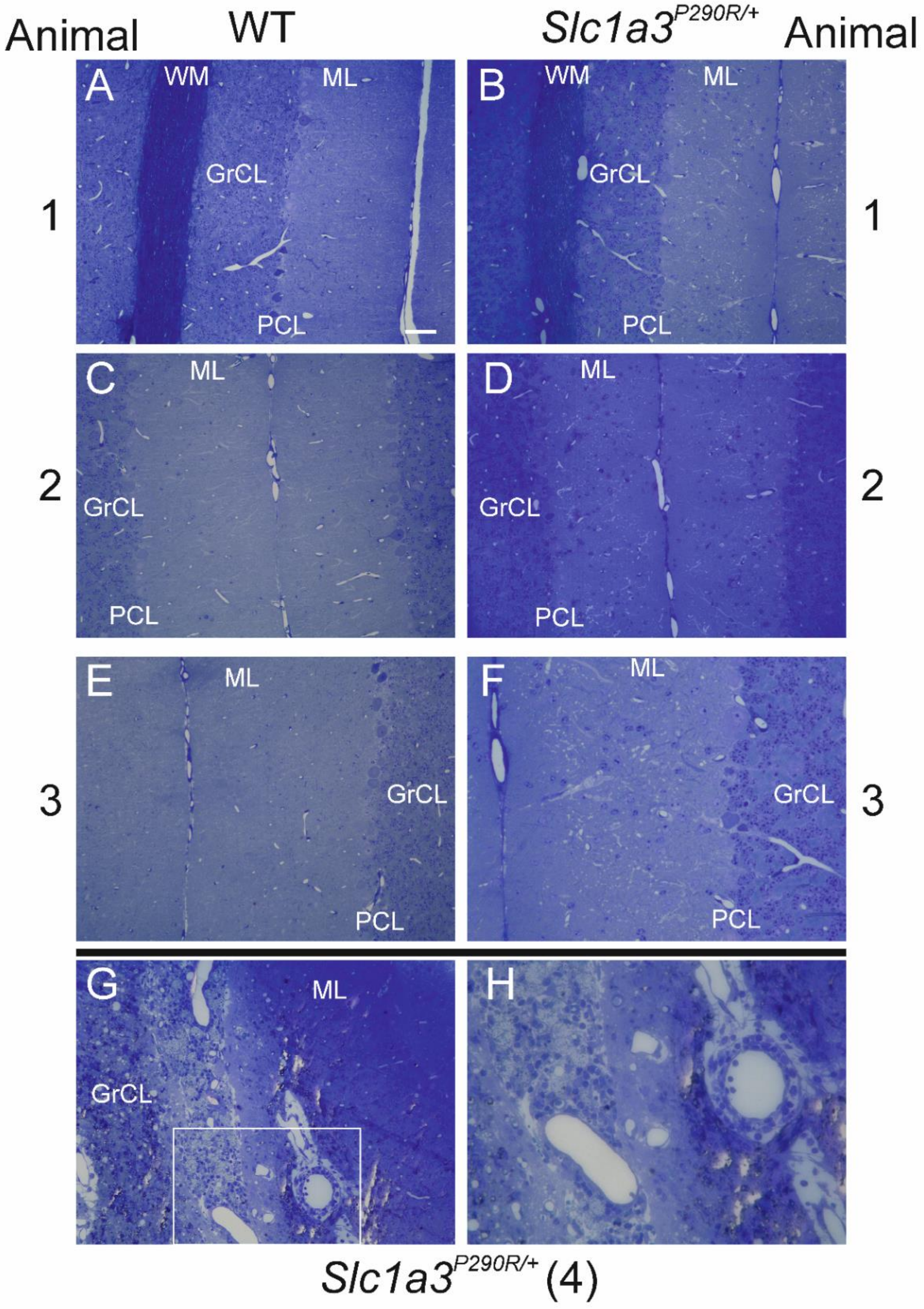

Supplementary Figure 5 – Semisections from the cerebella of WT and *Slc1a3*$^{P290R/+}$ animals at P50. A–F Nissl-stained sagittal semisections from cerebella in three WT (A, C, E) and *Slc1a3*$^{P290R/+}$ (B, D, F) mice, showing more nuclei in the molecular layer of cerebella in *Slc1a3*$^{P290R/+}$ mice. Nissl-stained sagittal section from a cerebellum of a fourth *Slc1a3*$^{P290R/+}$ mouse (**G**, **H**) shows a more severe cellular phenotype at this age. Magnified inset (H) of G. Scale bars: 50 µm (A–D, G), 25 µm (E, F, H).

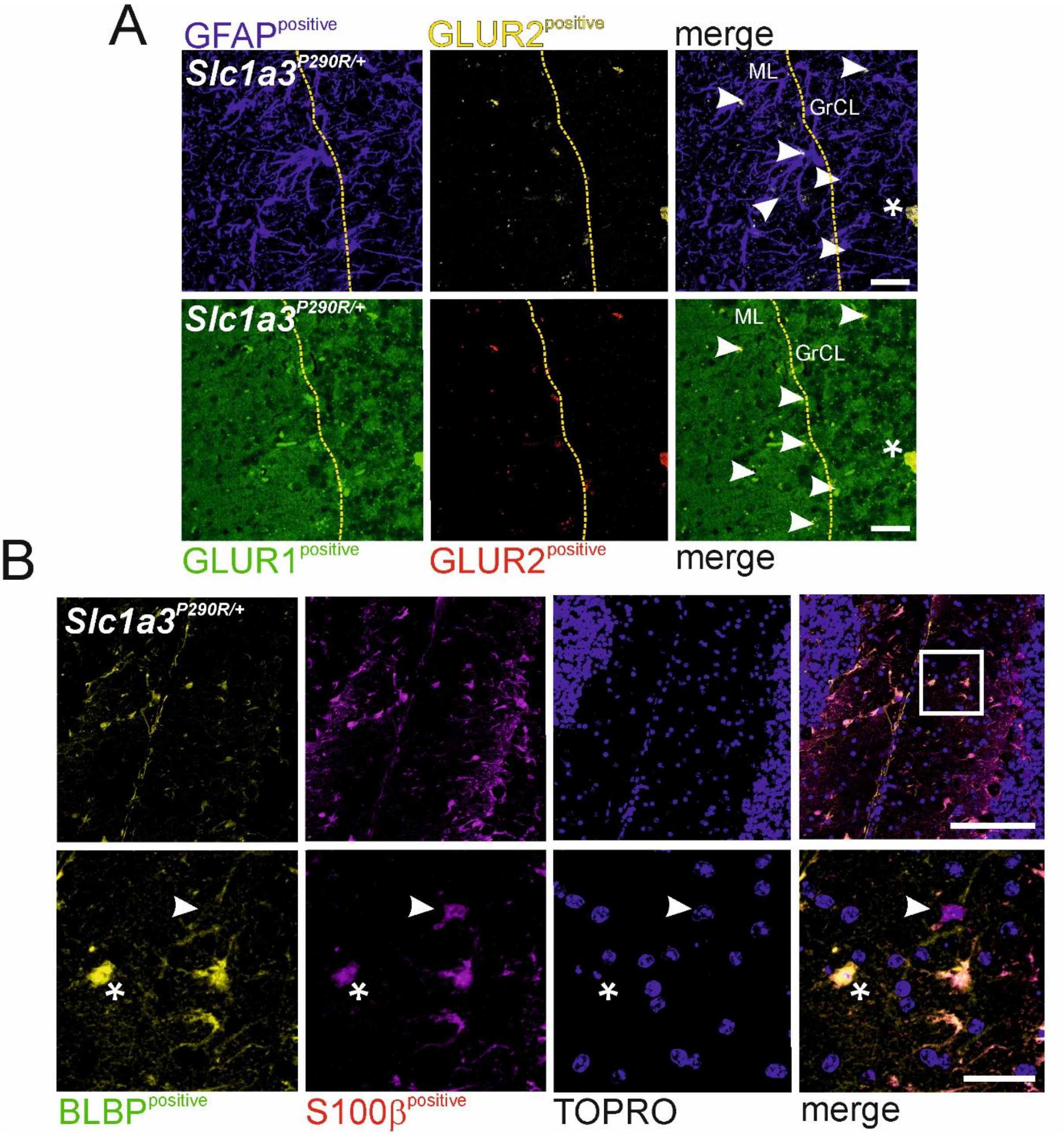

Supplementary Figure 6 – Glial cell types in the molecular layer of *Slc1a3*$^{P290R/+}$. Colocalization (**A**, top) of GFAP and GLUR2 is shown as white (arrowheads). (**A**, bottom) Colocalization of GLUR1 and GLUR2 is shown as yellow (arrowheads). Scale bar: 25 µm. Incidence of two different populations (**B**, BLBP^{positive}/S100β^{positive} (asterisks), BLBP^{negative}/S100β^{positive} (arrowheads)) of glial cells in the molecular layer from *Slc1a3*$^{P290R/+}$ mice. Scale bars 25 µm (A), 100 µm (top), 25 µm (bottom, B).

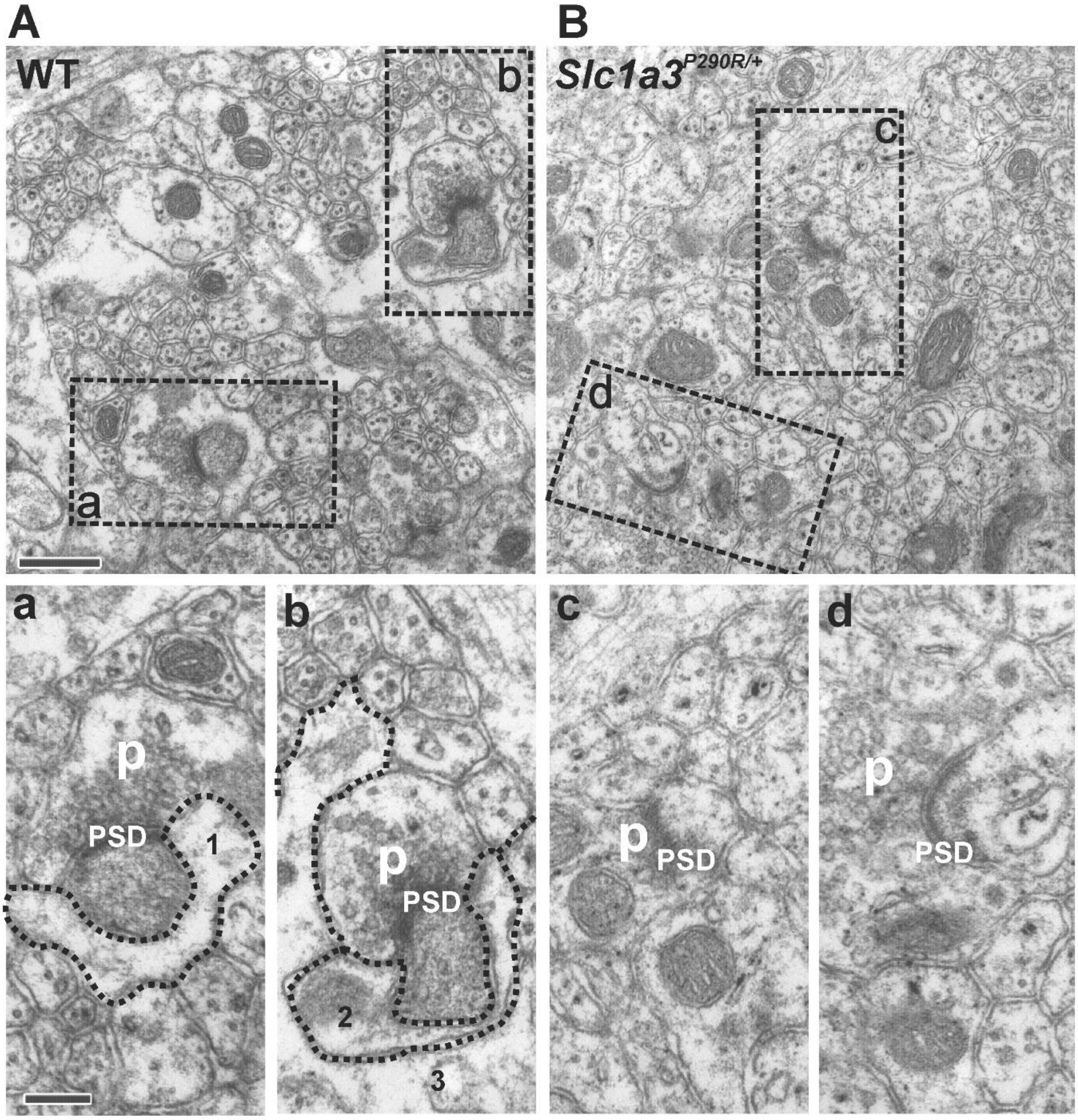

Supplementary Figure 7 – Electron micrographs show impaired wrapping of synapses in the molecular layer through glial cells in *Slc1a3*$^{P290R/+}$ samples. Overview with typical synapse morphology in a molecular layer from a WT mouse (**A**). Magnified insets of two representative regions (**a**, **b**) show the postsynaptic densities (PSD) and the presynapses (p). Dotted lines refer to glial cells, which are involved in shielding the synapse (1–3; bottom). Overview of an area in the molecular layer from a *Slc1a3*$^{P290R/+}$ mouse (**B**). Glial wrapping is virtually absent in *Slc1a3*$^{P290R/+}$ mice as shown in the magnified insets (**c**, **d**). Scale bars: 500 nm (A, B), 200 nm (a – d).

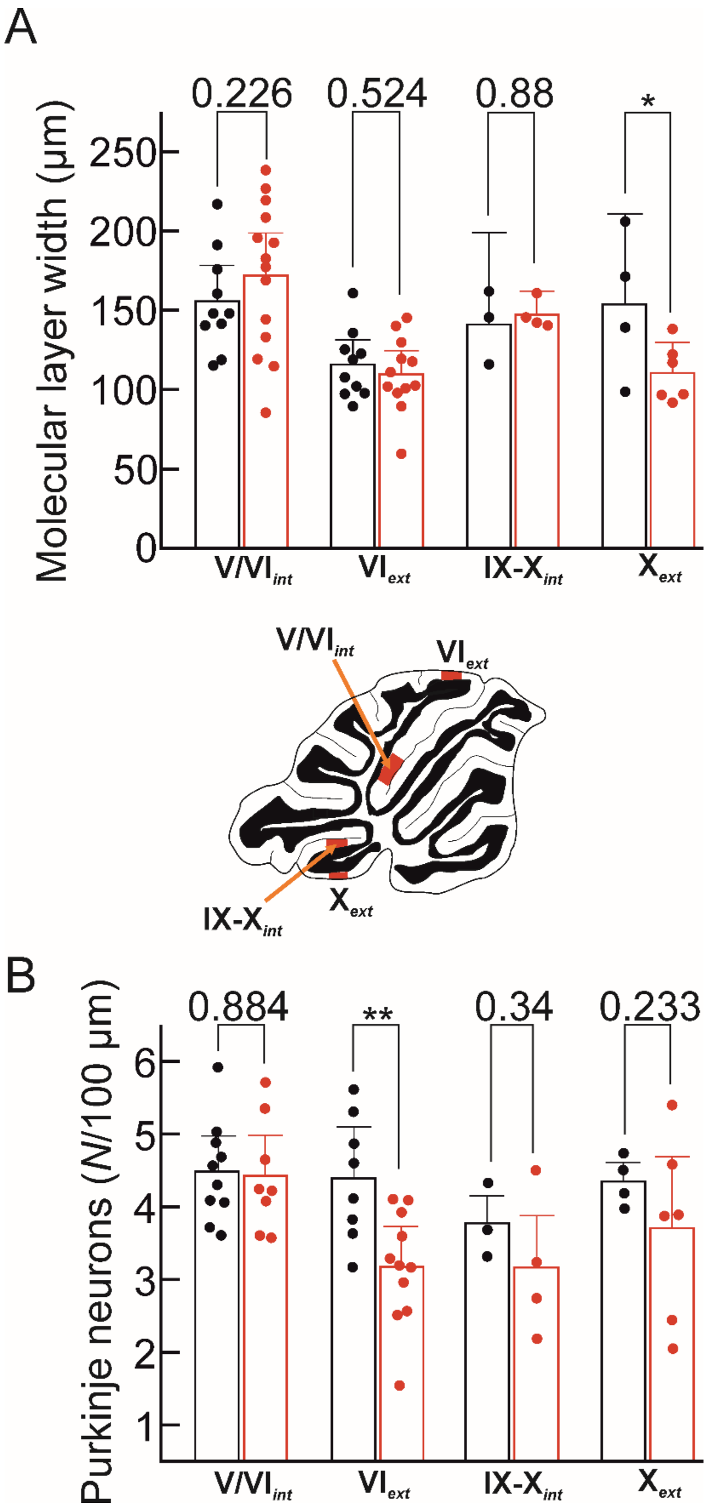

Supplementary Figure 8 – Statistical analysis of molecular layer thickness and Purkinje neuron numbers in WT and *Slc1a3*$^{P290R/+}$ animals. Bar graphs showing mean thicknesses of molecular layers (±*CI*) in the indicated regions (**A**) in the inset (middle). Bar graphs showing mean numbers (±*CI*) of Purkinje neurons/100 µm layer (**B**) in the indicated regions; WT (black), *Slc1a3*$^{P290R/+}$ (red); all points represent the values obtained from individual animals, respectively (A, B: 2-*way* ANOVA with Holm-Sidak *post hoc* tests); all *P*–values are available online for this figure (Supplementary Table 3).
