# Supplementary material for: Increased glutamate transporter-associated anion currents cause glial apoptosis in episodic ataxia 6
Source: Brain Commun. 2020 Mar 4;2(1):fcaa022. doi: 10.1093/braincomms/fcaa022 (PMC7425361; doi:10.1093/braincomms/fcaa022)
Supplement: fcaa022_Supplementary_Data [file fcaa022_supplementary_data.zip › Supplementary_Material.pdf]

Supplementary Material

for

Increased glutamate transporter-associated anion currents cause glial apoptosis in episodic ataxia 6

Peter Kovermann[1], Verena Untiet[1,3], Yulia Kolobkova[1], Miriam Engels[1], Stephan Baader[2], Karl Schilling[2], and Christoph Fahlke[1]

[1]Institut für Biologische Informationsprozesse, Molekular- und Zellphysiologie (IBI-1), Forschungszentrum Jülich, 52425 Jülich, Germany

[2]Anatomisches Institut, Rheinische Friedrich-Wilhelm-Universität, 53115 Bonn, Germany

[3]Center for Translational Neuromedicine, Københavns Universitet, 2200 København N, Denmark

Table of content for Supplementary Methods and Tables:

1. **Supplementary Methods**
   - 1.1 Transgenic animals, p. 2.
   - 1.2 Analysis of motor coordination of transgenic mice, p. 2.
   - 1.3 Visualization and quantification specific cell types and components, p. 4.
   - 1.4 Quantification of Bergmann glia cells, p. 5.
   - 1.5 Quantification of glutamatergic and GABAergic synapses, p. 5.
   - 1.6 Transmission electron microscopy, p. 5
   - 1.7 Terminal *d*UTP nick end labeling (TUNEL), p. 6.
   - 1.8 Western blot analyses, p. 6.
   - 1.9 Analysis of Purkinje neuron spiking and spike pauses, p. 7.
   - 1.10 Quantification of cerebellar degeneration in old animals, p. 8.
   - 1.11 Analysis of molecular layer width and Purkinje neuron numbers, p. 8.
   - 1.12 Supplementary References, p. 8.

2. **Supplementary Tables**
   - 2.1 Supplementary Table 1: Score sheet and stop criteria for animal breeding and animal testing, p. 9.
   - 2.2 Supplementary Table 2: Details about used antibodies, p. 10.
   - 2.3 Supplementary Table 3: *P*-values for figures, p. 11–19.

1. Supplementary Methods

1.1 Transgenic animals

Knock-in *Slc1a3*^{tm1P290RCfa} (*Slc1a3*$^{P290R/+}$) mice were generated by site-directed mutagenesis using a mutant *Slc1a3* allele located on chromosome 15 (at 3.82 cM) and encoding a P290R substitution in exon 7. Briefly, a fragment covering exons 4–8 of the *Slc1a3* locus was subcloned and the P290R sequence (agg to gcg) was inserted via PCR synthesis into the end of exon 7 (Polygene Transgenetics, Switzerland). Positive clones were injected into blastocysts from C57BL/6N mice, and surviving blastocysts were the transferred to a CD–1 foster mouse. Germline chimeric foster mice were crossbred with C57BL/6N-derived FLP-deleter mice (B6;SJL-Tg(ACTFLPe)9205Dym/J; JANVIER LABS, France) to excise the neomycin cassette (Supplementary Fig. 1). Since mortality of C57BL/6 knock-in mice prevented successful breeding, we backcrossed the mutation into the more robust mouse strain 129/SvJ (Taconic Biosciences GmbH, Germany) for 10 generations. The animals were housed under standard conditions in the animal facility of Forschungszentrum Jülich according to institute guidelines under a 12 h light/dark cycle (light: 6:00am–6:00pm). Animal testing was done during the light phase. All experiments complied with the German Law for the Protection of Animals and were approved by the Forschungszentrum Jülich and LANUV (State Agency for Nature, Environment and Consumer Protection) of North Rhine-Westphalia (reference nos 84–02.04.2014.A334/A335, to P.K.). The health status of mutant animals was continuously monitored, and animals were treated in strict accordance with defined stop criteria (Supplementary Table 1).

1.2 Analysis of motor coordination of transgenic mice

Motor coordination of transgenic animals was tested with two different tests. We used ledge tests (Guyenet *et al.*, 2010) to test the behavior of animals on a narrow path.

Animals were placed on a 1 cm wide ledge and the test were video-recorded for analysis. Mice younger than postnatal day 30 (P30) were not subjected to the test because they tended be more active (e.g. jumpiness) (van Abeelen and Schoones, 1977; Curzon, 2009). The mean stumble ratio was calculated from the video recordings as the average error rate for each animal during all traverses across the ledge using the equation:

$$\boldsymbol{stumble\ ratio} = \frac{\Sigma_1^n \left(N_{failures} \cdot N_{steps}^{-1}\right)_1}{n} \text{ (Eq. 1).}$$

n = number of traverses (2´); $N_{failures}$ = number of paw slips/traverse; N_{steps} = number of steps/traverse.

Mice were as well subjected to another test of motor coordination, the rotarod test. This test measures the time until an animal falls from a rotating rod. The rotarod sessions were video-recorded and then analyzed by latencies to fall and counting spin failures. For each fall event, the remaining score (time in sec) was subtracted from the starting value of 600 (~ test period: 600 s), and the resulting value was corrected for spin failures (Supplementary Fig. 3) by subtracting three points for each missed cycle. Spin failure frequencies for WT and mutant mice were calculated from the number of spin failures during latency to fall. We assumed a lower limit of 1/600 s (≈0.0017 Hz, Fig. 1E). In initial tests we observed that mutant males increase their latency to fall from the rod via excessive spin failures, preventing an appropriate comparison of WT and mutant mice. We therefore excluded males from rotarod tests. Time series data of rotarod tests were averaged and transformed into survival plots (Kaplan, 1958), and corrected latencies were added as box plots (Fig. 1D and F). Blinding and randomization was not possible because of the severe phenotype of mutant animals. The State Agency for Nature, Environment and Consumer Protection of North Rhine-Westphalia (reference nos 84–02.04.2014.A334/A335, to P.K.) ordered permanent

observation of transgenic mice during breeding and maintenance. Tests were always performed with all litters, the group sizes were determined by genotype frequency (50:50). All data points in Fig. 1C, and F represent mean values obtained from individual animals during 3 trials per session.

1.3 Visualization and quantification of specific cell types and components

Mice were anesthetized with isoflurane (Piramal Healthcare, UK) and decapitated. The cerebella were excised, transferred to 4% paraformaldehyde (PFA, $[CH_2O]_n$) in phosphate buffer (PB; in mM, 81 $NaH_2PO_4 \cdot 2H_2O$, 9 $Na_2HPO_4H_2O$, pH 7.4), and stored in fixative for 5 days (at 4°C). After dehydration in a graded ethylalcohol series (50%, 70%, 90%, 100% EtOH) brains were embedded in paraffin and sagittal sections (5 µm or 7 µm thick) were cut for immunostainings. Nissl staining was performed as previously described (Paul *et al*, 2008). For immunostaining, endogenous peroxidases were inactivated (in mM, 21 $C_6H_8O_7$ and 6 Na_2HPO_4, 15% (v/v) H_2O_2 for 15´, at RT), and epitopes were unmasked (2000 Tris and 63 EDTA, pH 8.0, at 100°C for 20´) and blocked overnight (bovine serum albumin 1% (w/v) in PB, at 4°C). Sections were incubated for 1-2 days with primary and 45-60´with secondary antibodies diluted in CTA (i.e. 5%ChemiBLOCKER, Merck–Millipore, in PB containing 1% Triton-X100 and 0.05% NaN_3) and 1% Triton-X100. For visualization, sections were incubated in cyanine-dye conjugated secondary antibodies for 60´ at room temperature (RT). A list of all used antibodies is given in in Supplementary Table 2. Fiber or cell numbers in WT and mutant cerebella was counted in sagittal vermis sections (lobes V*/*VI and IX*/*X). Bergmann glia cells were quantified by counting the number of $GFAP^{positive}$ fibers in the molecular layer (ML) and the density of neurons or astrocytes by counting the number of $NeuN^{positive}$ cells or $GFAP^{positive}$ cells with a typical multipolar astrocyte shape in defined areas.

1.4 Quantification of Bergmann glia cells

We immunostained paraffin slices (4 µm) from mice cerebella with typical astrocytic and Bergmann glial markers as brain lipid binding protein (BLBP), the Ca^{2+} binding protein S100β, and glutamate-aspartate transporter (GLAST) and counterstained nuclei with TOPRO-3 for confocal microcopy. Bergmann glial cell somata in the Purkinje cell layer (PCL) and ectopic glia in the molecular layer (ML, lobes V/VI and IX/X) were counted separately. Cell numbers are provided as mean numbers of Bergmann glia per 100 µm length (PCL or ML) from individual animals.

1.5 Quantification of glutamatergic and GABAergic synapses in the molecular layer

To determine the number of synapses in the cerebellar molecular layer, paraffin slices were incubated overnight with a combination of primary antibodies against vesicular glutamate transporter 1, vesicular glutamate transporter 2, and anti-glutamate decarboxylase 65/67 and subsequently in cyanine-dye conjugated secondary antibodies from donkey for 60´ at RT. Densities of synapses were quantified by counting $VGLUT1^{positive}$, $VGLUT2\text{-}^{positive}$ and $GAD65/67^{positive}$ signals within single confocal planes in randomly selected areas of different sizes of V/VI_{int}, VI_{ext}, IX/X_{int}, and X_{ext}. Synapse density was calculated in n = 3-5/4-9 (WT/Mut) mice from means of n = 144/212 (WT/Mut) test areas. Densities are given as boutons per 100 $µm^2$.

1.6 Transmission electron microscopy

Electron microscopical pictures (Supplementary Fig. 7) were prepared as described previously (Miething, 1992; Jankowski *et al.*, 2009). Briefly, mice were perfused with Karnovsky solution (3% PFA), 3% glutaraldehyde ($C_5H_8O_2$), in phosphate buffered saline containing 10 mM $NaH_2PO_4 \cdot H_2O$ and 150 mM NaCl, pH 7.2) and dissected cerebella were immersed in the same fixative for 6 h. Tissue blocks (1 mm thick) were

cut and incubated in 1% Osmiumtetroxid (OsO_4) at RT for 120´. Sections were then dehydrated and embedded in Epon 812 using a 3:2 mixture of Epon A (38% Glycidether 100 in 2-Dodecenylsuccinic acid anhydride, $C_{16}H_{26}O_3$) and Epon B (53% Glycidether 100 in Methylnadic anhydride, $C_{10}H_{10}O_3$). After hardening, semithin sections (1 µm thick) were cut by a Ultramicrotome (Ultracut E from Reichert-Jung) and stained with a 4:1 solution of toluidine blue: pyronine G. Ultrathin sections (70 nm thick) were cut and mounted on Formvar coated slot grids (Plano, Wetzlar, Germany). Grids have been air dried and stained in 2% uranyl acetate and 0.5% lead citrate each for 10´. Sections were analyzed with an electron microscope (EM910, Carl Zeiss, Jena, Germany) equipped with an integrated digital camera (TRS 1K, Carl Zeiss).

1.7 Terminal dUTP nick end labeling (TUNEL)

We used the BrdU-Red DNA Fragmentation assay kit (Abcam, ab66110) for visualization of fragmentated DNA in Bergmann glia nuclei with the TUNEL method. Cryo slices from fixed cerebella (4% PA) from GFAP-EGFP expressing mice were permeabilized overnight at RT with CTA (ChemiBLOCKER, Merck-Millipore) and washed thoroughly with phosphate buffer (PB). Terminal deoxynucleotidyl transferase (T*d*T) enzymes were applied with bromolated *d*UTP for 60´ at 37°C in the dark. Cells were stained with BrdU-Red (Ex/Em: 488/576 nm) labeled antibodies (30´, RT) for visualization of TUNEL signals and with TOPRO-3 (1/1000) for DNA counterstaining. Cerebellar slices were imaged with confocal microscopy within 180´ after staining procedure. All data points in Fig. 5E represent mean values obtained from individual animals.

1.8 Western blot analyses

To quantify relative protein expression levels of the Bergmann glial marker proteins GLAST (glutamate-aspartate transporter), S100β, and BLBP (brain lipid binding

protein, Supplementary Fig. 4), we performed western blots from whole cerebellar lysates. Briefly, 3-4 mice were decapitated and their cerebella were homogenized and centrifuged (4°C) at 100,000 g (45´). Supernatant and pellet were separated and individual processed and run on SDS-PAGEs. Transfer of proteins to PVDF membrane was achieved on a Semi-dry blotter (V20-SDB, Scie-Plas) and proteins were visualized with antibodies listed in Supplementary Table 2. Blots were analyzed with Fiji (NIH, USA).

1.9 Analysis of Purkinje neuron spiking and spike pauses

Mean CV2 values from Purkinje neuron spiking activity were calculated using the following equation (Holt *et al.*, 1996):

$$\text{mean of ISI CV2} = \frac{1}{n}\sum_{i=1}^{n} 2 \cdot \frac{|ISI_{i+1} - ISI_i|}{(ISI_{i+1} + ISI_i)} \text{(Eq. 3).}$$

Interspike intervals (ISIs) in burst plots (Fig. 6B and D) are shaded according to their temporary non-averaged CV2 values during ISI_i to ISI_{i+1}. We defined burst initiation and termination by the lengths of the preceding and successive ISIs, respectively, corrected for the spike activity of each analyzed cell. An ISI of at least the mean ISI + 3σ in each cell was defined as a gap between bursts. Purkinje neuron spiking frequencies were determined within these individual bursts (Fig. 6). CF pauses were evoked by brief (200 ms) pressure ejections of bath solution supplemented with 1 mM Na-glutamate to molecular layer areas (Fig. 7), distal to the Purkinje cell layer with approximately 50−75 µm distance to Purkinje cell somata (PDES-DXH: npi electronic GmbH, 71732 Tamm, Germany). The recovery times were defined as the silent periods between glutamate puffs, and induced bursting activity and recovery of pre-puff simple spike activity. All data points in Fig. 6 and Fig. 7 represent mean values obtained from individual animals.

1.10 Quantification of cerebellar degeneration in old animals

Paraffinated cerebella (n = 10/10 animals, WT/Mut, five males and five females for both genotypes) were placed on a grid surface (grid size: 0.25 mm^2) and the dorsal surfaces were measured (Fiji Software, National Institutes of Health, USA). Degeneration in sliced vermis sections was quantified by estimating sagittal area sizes (n = 4/4 animals, WT/Mut) after Nissl staining for the white matter, granule cell layer, and molecular layer areas (Fig. 8).

1.11 Analysis of molecular layer width and Purkinje neuron numbers

The thickness of molecular layers was measured with Fiji (ImageJ) in the same regions (n = 3-10/4-14, WT/Mut) as indicated in Supplementary Fig. 8A. The numbers of Purkinje neurons in younger animals (P27−60) were counted in the internal/external vermal areas V/VI_{int}, VI_{ext}, IX/X_{int}, and X_{ext} and given as numbers of identified Purkinje neurons per 100 µm length of the Purkinje neuron layer (n = 3-10/4-11 animals, WT/Mut (Supplementary Fig. 8B).

1.12 Supplementary References

Curzon P, Zhang, M., Radek, R.J., Fox, G. B. The Behavioral Assessment of Sensorimotor Processes in the Mouse: Acoustic Startle, Sensory Gating, Locomotor Activity, Rotarod, and Beam Walking. In: Buccafusco JJ, editor. *Methods of Behavior Analysis in Neuroscience 2nd edition.* Boca Raton (FL); 2009: 146.

Guyenet SJ, Furrer SA, Damian VM, Baughan TD, La Spada AR, Garden GA. A simple composite phenotype scoring system for evaluating mouse models of cerebellar ataxia. *J Vis Exp* 2010, 39.

Holt GR, Softky WR, Koch C, Douglas RJ. Comparison of discharge variability in vitro and in vivo in cat visual cortex neurons. *J Neurophysiol* 1996; 75(5): 1806-14.

Jankowski J, Miething A, Schilling K, Baader SL. Physiological purkinje cell death is spatiotemporally organized in the developing mouse cerebellum. *Cerebellum* 2009; 8(3): 277-90.

Kaplan EL, Meier, P. Nonparametric estimation from incomplete observations. *J Am Ass* 1958; 53: 34.

Miething A. Ultrathin sectioning of different areas of the same semithin section. *Microscopy Res Techniq* 1992; 21(1): 73-4.

Paul CA, Beltz B, Berger-Sweeney J. The nissl stain: A stain for cell bodies in brain sections. *CSH Protoc* 2008, prot 4806.

van Abeelen JH, Schoones AH. Ontogeny of behavior in two inbred lines of selected mice. *Dev Psychobiol* 1977; 10(1): 17-23.

2. Supplementary Tables

2.1 Supplementary Table 1:

Score sheet and stop criteria for breeding and animal experiments for mouselines *Slc1a3*^{tm1P290RCfa} (KI P290R Slc1a3) and *Slc1a3*^{tm1Kta} (GLAST KO).

**Observation:**	**Score**
**1. Body weight:**	
correlates with mean body weight of particular age	**0**
decrease: ≤ 5% of mean body weight of particular age	**1**
decrease: 5 – 10% of mean body weight of particular age	**2**
decrease: 11 – 20% of mean body weight of particular age	**3**
decrease: >20% of mean body weight of particular age	**4**
**2. General condition:**	
smooth and glossy fur; clean orifices of the body, clear, glossy eyes,	**0**
defects in fur (overwhelming or reduced grooming)	**1**
unordered and or dead fur, impure orifices of the body	**2**
dead/impure fur, clotted orifices of the body, dreary eyes	**3**
clear breath sounds, animal feel cold, seizures	**4**
**3. Spontaneous behavior**	
Normal behavior (animal react on blowing/touches), normal social contacts.	**0**
slight problems in motor coordination, temporary tremor, normal social contacts	**1**
limited motor coordination, clear, rare rearing and climbing	**2**
Isolation, lethargy and curved posture	**3**
Isolation, lethargy and curved posture with frequent seizures (>1/8h) or longer than 2´	**4**
**4. Symptomatik (Episodic ataxia)**	
no symptoms	**0**
Slight ataxia, food and water is reached easily, temporary tremor	**1**
clear ataxia, tremor and short focal seizures, w/o loss of righting reflex, frequency <1/8h, food and water is consumed	**2**
Heavy ataxia, short focal seizures, w/o loss of righting reflex (<2/8h), with relaxing phase.	**3**
Heavy ataxia with frequent focal seizures and loss of righting reflex for >2´ or frequency of seizures >1/day	**4**

2.2 Supplementary Table 2

Target	full name	host	RRID	Use/dilution	Supplier
1st antibodies					
BLBP	brain lipid binding protein	*rb*	AB_10000325	1:200	MerckMillipore, Germany
CASP3	active caspase-3	*rb*	AB_302962	1:20	Abcam, UK
GAD65/67	glutamate decarboxylase 65/67	*rb*	AB_477019	1:5,000	Sigma, Germany
GFAP	glial fibrillary acidic protein	*ms*	AB_477010	1:800	Sigma, Germany
GLAST	glutamate aspartate transporter	*ms*	AB_10829302	1:500	Milteniy, Biotec, Germany
GLUR1	glutamate receptor 1	*gt*	AB_641039	1:100	Santa Cruz, USA
GLUR2	glutamate receptor 2	*rb*	AB_2313803	1:100	MerckMillipore, Germany
NeuN	neuronal nuclei	*rb*	AB_104225	1:400	Abcam, UK
S100	S100β	*ms*	AB_301508	1:100	Abcam, UK
VGLUT1	vesicular glutamate transporter 1	*ms*	AB_887875	1:100	Synaptic Systems, Germany
VGLUT2	vesicular glutamate transporter 2	*gp*	AB_2301731	1:10,000	Merck-Millipore, Germany
2nd antibodies					
Cy2	cyanidin 2-conjugated antibody	*d@rb*	AB_2340612	1:400	Dianova, Germany
Cy2	cyanidin 2-conjugated antibody	*d@gt*	AB_2307341	1:400	Dianova, Germany
Cy2	cyanidin 2-conjugated antibody	*d@ms*	AB_2340827	1:400	Dianova, Germany
Cy3	cyanidin 3-conjugated antibody	*d@gp*	AB_2340460	1:800	Dianova, Germany
Cy5	cyanidin 5-conjugated antibody	*d@ms*	AB_2340813	1:400	Dianova, Germany
Cy5	cyanidin 5-conjugated antibody	*d@rb*	AB_2340607	1:400	Dianova, Germany
Assay					
TOPRO-3	TOPRO-3		No. T3605	1:1,000	Invitrogen, Germany
TUNEL Labeling Kit - BrDU-Red (DNA fragmentation)			No. ab66110	NA	Abcam, UK

rb - rabbit; *ms* - mouse; *gt* - goat; *gp* - guinea pig; *d* - donkey
RRID Portal (Resource identification portal): *https://scicrunch.org/resources*

2.3 Supplementary Table 3: *P-values for Figures*

Figure 1C (median paw slips per step during the ledgetests)

2-way ANOVA	Significance α = 0.05/0.1?	Summary	*P*-value[Holm-Sidak]
WT::P290R/+	YES	***	<0.001
WT: ♂::♀	NO	*n.s.*	0.441
P290R/+: ♂::♀	NO	*n.s.*	0.124
♂: WT::P290R/+	YES	***	<0.001
♀: WT::P290R/+	YES	***	<0.001

Figure 1E (mean frequencies of spin failures on the rotarod)

2-*way* ANOVA*repeated measures*	Significance α = 0.05/0.1?	Summary	P-value*Holm-Sidak*
WT::P290R/+ *Trial I*	YES	***	<0.001
WT::P290R/+ *Trial II*	YES	**	0.005
WT::P290R/+ *Trial III*	NO/YES	*n.s.*	0.097

Calculation was performed using average latencies of individual animals from the three trials, respectively

Figure 1F (median rotarod latencies)

2-*way* ANOVA*repeated measures*	Significance α = 0.05/0.1?	Summary	P-value*Holm-Sidak*
WT::P290R/+ *Trial I*	YES	**	0.01
WT::P290R/+ *Trial II*	YES	*	0.031
WT::P290R/+ *Trial III*	NO	*n.s.*	0.359

Calculation was performed using average latencies of individual animals from the three trials, respectively

Figure 2B (mean numbers of GFAP fibers per 100 µm PCL in region V/VI)

2-way **ANOVA**	**Significance α = 0.05/0.1?**	**Summary**	***P*-value** [Holm-Sidak]
WT::P290R/+ (Age 1)	NO	*n.s.*	0.784
WT::P290R/+ (Age 2)	YES	*	0.012
WT::P290R/+ (Age 3)	NO	*n.s.*	0.371
WT::P290R/+ (Age 4)	YES	**	0.005
WT::P290R/+ (Age 5)	YES	***	<0.001
WT::P290R/+ (Age 6)	YES	***	<0.001
WT::P290R/+ (Age 7)	YES	***	<0.001
WT::P290R/+ (Age 8)	YES	***	<0.001

Figure 2B (mean numbers of GFAP fibers per 100 µm PCL in region IX/X)

2-way **ANOVA**	**Significance α = 0.05/0.1?**	**Summary**	***P*-value** [Holm-Sidak]
WT::P290R/+ (Age 1)	NO	*n.s.*	0.795
WT::P290R/+ (Age 2)	NO/YES	*n.s.*	0.099
WT::P290R/+ (Age 3)	NO	*n.s.*	0.318
WT::P290R/+ (Age 4)	YES	*	0.018
WT::P290R/+ (Age 5)	YES	***	<0.001
WT::P290R/+ (Age 6)	YES	***	<0.001
WT::P290R/+ (Age 7)	YES	***	<0.001
WT::P290R/+ (Age 8)	YES	***	<0.001

Age groups: P5-6 (1), P8-9 (2), P10 (3), P11-12 (4), P15-20 (5), P24-P35 (6), P45-65 (7), P538-P950 (8)

Figure 2C (median BGC^{+} cells/100 µm PCL in region V/VI)

2-way **ANOVA**	**Significance α = 0.05/0.1 ?**	**Summary**	***P*-value$^{\text{Holm-Sidak}}$**
WT::P290R/+ (PCL)	YES	***	<0.001
WT::P290R/+ (ectopic)	NO	*n.s.*	0.185

Figure 2C (median BGC^{+}cells/100 µm PCL in region IX/X)

2-way **ANOVA**	**Significance α = 0.05/0.1 ?**	**Summary**	***P*-value$^{\text{Holm-Sidak}}$**
WT::P290R/+ (PCL)	YES	***	<0.001
WT::P290R/+ (ectopic)	NO	*n.s.*	0.298

Figure 2F (median NeuN^{+} cells /mm^{2})

2-way **ANOVA**	**Significance α = 0.05/0.1 ?**	**Summary**	***P*-value$^{\text{Holm-Sidak}}$**
WT::P290R/+ (P20–P40)	YES	**	0.002
WT::P290R/+ (P41-100)	YES	*	0.025
WT::P290R/+ (>P100)	YES	***	0.001

Figure 2G (median GFAP^{+} cells/mm^{2})

2-way **ANOVA**	**Significance α = 0.05/0.1 ?**	**Summary**	***P*-value$^{\text{Holm-Sidak}}$**
WT::P290R/+ (P20–P40)	NO/YES	*n.s.*	0.093
WT::P290R/+ (P41–100)	NO	*n.s.*	0.121
WT::P290R/+ (>P100)	YES	**	0.002

**Figure 3B (median VGLUT1⁺ clusters /100 µm²)**			
***2-way* ANOVA**	**Significance α = 0.05/0.1 ?**	**Summary**	***P*-value$^{Holm\text{-}Sidak}$**
WT::P290R/+ (V/VI)	YES	***	<0.001
WT::P290R/+ (VI)	YES	**	0.006
WT::P290R/+ (IX/X)	YES	**	0.003
WT::P290R/+ (X)	YES	***	<0.001

**Figure 3C (median VGLUT2⁺ clusters /100 µm²)**			
***2-way* ANOVA**	**Significance α = 0.05/0.1 ?**	**Summary**	***P*-value$^{Holm\text{-}Sidak}$**
WT::P290R/+ (V/VI)	YES	***	<0.001
WT::P290R/+ (VI)	YES	*	0.049
WT::P290R/+ (IX/X)	YES	***	<0.001
WT::P290R/+ (X)	YES	*	0.015

**Figure 3D (median GAD65/67⁺ clusters /100 µm²)**			
***2-way* ANOVA**	**Significance α = 0.05/0.1 ?**	**Summary**	***P*-value$^{Holm\text{-}Sidak}$**
WT::P290R/+ (V/VI)	YES	*	0.046
WT::P290R/+ (VI)	NO	*n.s.*	0.123
WT::P290R/+ (IX/X)	NO	*n.s.*	0.595
WT::P290R/+ (X)	NO	*n.s.*	0.867

Figure 4A (mean current amplitudes, pA)

***U*-test**	**Significance α = 0.05/0.1 ?**	**Summary**	***P*-value**
WT::P290R/+	YES	*	0.024

Figure 4C (normalized current amplitudes at -100/-120 mV, pA)

2-way ANOVA(repeated measures)	**Significance α = 0.05/0.1 ?**	**Summary**	***P*-value**
WT::P290R/+ (-100 mV)	YES	*	0.025
WT::P290R/+ (-120 mV)	YES	*	0.017
WT, -80 mV::-100/120 mV	NO/NO	*n.s./n.s.*	0.628/0.282
P290R/+, -80 mV::-100/-120 mV	YES/YES	*/**	0.024/0.004

Figure 4E (Comparisons WT :: P290R: $[Cl^-]_{int}$, mM)

***2-way* ANOVA**	**Significance α = 0.05/0.1 ?**	**Summary**	***P*-value$^{Holm-Sidak}$**
WT::P290R/+ (P4-7)	YES	***	<0.001
WT::P290R/+(P8-10)	YES	***	<0.001
WT::P290R/+(P11-12)	YES	***	<0.001
WT::P290R/+(P13-15)	NO/YES	*n.s.*	0.09

Figure 4 F (Comparisons WT :: P290R+UCPH101: $[Cl^-]_{int}$, mM)

***1-way* ANOVA on ranks**	**Significance α = 0.05 ?**	**Summary**	**Dunn´s test**
WT::P290R/+(P4-12)	YES	***	YES
WT::WT-P290R/+^{UCPH}(P4-12)	NO	*n.s.*	NO

1-way ANOVA on ranks WT::P290R/+: *P* < 0.001

Figure 5B (left, total apoptotic events)

2-way ANOVA	Significance α = 0.05/0.1 ?	Summary	*P*-value[Holm-Sidak]
WT::P290R/+ (P8-9)	NO/YES	*n.s.*	0.074
WT::P290R/+ (P10)	YES	**	0.008
WT::P290R/+ (P11)	YES	*	0.043
WT::P290R/+ (P12)	YES	*	0.015
WT::P290R/+ (P13)	NO/YES	*n.s.*	0.073
WT::P290R/+ (P14-15)	NO	*n.s.*	0.56

Figure 5B (right, colocalized GFAP-EGFP/CASP3 signals)

2-way ANOVA	Significance α = 0.05/0.1 ?	Summary	*P*-value[Holm-Sidak]
WT::P290R/+ (P8-9)	NO/YES	*n.s.*	0.056
WT::P290R/+ (P10)	YES	*	0.038
WT::P290R/+ (P11)	YES	***	<0.001
WT::P290R/+ (P12)	YES	***	<0.001
WT::P290R/+ (P13)	YES	*	0.045
WT::P290R/+ (P14-15)	NO	*n.s.*	0.369

Figure 5E, (*left*, colocalized GFAP-EGFP/TUNEL signals)

1-way ANOVA on ranks	Significance α = 0.05/0.1 ?	Summary	Dunn´s test
WT::P290R/+ (P9-14)	YES	***	YES

1-way ANOVA on ranks WT::P290R/+: $P = 0.001$

Figure 5E, *right* (total apoptosis events, TUNEL signals)

1-way ANOVA on ranks	Significance α = 0.05/0.1 ?	Summary	Dunn´s test
WT::P290R/+ (P9-14)	YES	**	YES

1-way ANOVA on ranks WT::P290R/+: $P = 0.005$

**Figure 6E (spiking frequencies of PCs, Hz)**			
***2-way* ANOVA**	**Significance α = 0.05/0.1 ?**	**Summary**	***P*-value$^{\text{Holm-Sidak}}$**
WT::P290R/+ (P10)	NO	*n.s.*	0.331
WT::P290R/+ (P20–30)	NO	*n.s.*	0.442
WT::P290R/+ (P40)	YES	***	<0.001

**Figure 6F (covariances of PC spiking, CV)**			
***2-way* ANOVA**	**Significance α = 0.05/0.1 ?**	**Summary**	***P*-value$^{\text{Holm-Sidak}}$**
WT::P290R/+ (P10)	NO	*n.s.*	0.24
WT::P290R/+ (P20–30)	NO	*n.s.*	0.685
WT::P290R/+ (P40)	NO	*n.s.*	0.348

**Figure 6G (covariances of PC spiking, mCV2)**			
***2-way* ANOVA**	**Significance α = 0.05/0.1 ?**	**Summary**	***P*-value$^{\text{Holm-Sidak}}$**
WT::P290R/+ (P10)	NO	*n.s.*	0.242
WT::P290R/+ (P20–30)	NO	*n.s.*	0.605
WT::P290R/+ (P40)	NO	*n.s.*	0.383

**Figure 7C (CF-pauses of PCs, s)**			
***U*-test**	**Significance α = 0.05/0.1 ?**	**Summary**	***P*-value**
WT::P290R/+	YES	*	*P* = 0.014

Figure 8B (transversal cerebellar areas, mm^2)

2-way ANOVA	Significance α = 0.05/0.1 ?	Summary	*P*-value$^{Holm-Sidak}$
WT♂::P290R/+♂ (H_l)	NO/YES	*n.s.*	0.077
WT♂::P290R/+♂ (H_r)	NO	*n.s.*	0.177
WT♂::P290R/+♂ (V_a)	YES	*	0.032
WT♂::P290R/+♂ (V_p)	YES	***	<0.001
**Σ WT♂::P290R/+♂**	**YES**	**	**0.005**
WT♀::P290R/+♀ (H_l)	YES	***	<0.001
WT♀::P290R/+♀ (H_r)	YES	***	<0.001
WT♀::P290R/+♀ (V_a)	YES	**	0.004
WT♀::P290R/+♀ (V_p)	YES	***	0.001
**Σ WT♀::P290R/+♀**	**YES**	*******	**<0.001**

(H_l: left hemisphere; H_r: right hemisphere; V_a: anterior vermis; V_p: posterior vermis)

Figure 8D (sagittal cerebellar areas, vermis, mm^2)

2-way ANOVA	Significance α = 0.05/0.1 ?	Summary	*P*-value$^{Holm-Sidak}$
WT::P290R/+ (*WM*)	NO	*n.s.*	0.154
WT::P290R/+ (*GCL*)	YES	***	<0.001
WT::P290R/+ (*ML*)	YES	***	<0.001
**Σ WT::P290R/+ (*Vermis*)^t**	**YES**	**	**0.002**

(WM: white matter; GCL: granule cell layer; ML: molecular layer)

Supplementary Figure 3B (latencies to first error, s)

2-way ANOVA (*repeated measures)*	Significance α = 0.05/0.1 ?	Summary	P-value[Holm-Sidak]
WT::P290R/+ *Trial I*	YES	*	0.01
WT::P290R/+ *Trial II*	YES	**	0.005
WT::P290R/+ *Trial III*	YES	**	0.004

Supplementary Figure 4D (relative protein expression, normalized)

2-way ANOVA	Significance α = 0.05/0.1 ?	Summary	*P*-value[Holm-Sidak]
WT::P290R/+ (BLBP)	YES	**	0.004
WT::P290R/+ (S100β)	YES	**	0.007
WT::P290R/+ (GLAST)	YES	***	<0.001

Supplementary Figure 8A (width of ML, µm)

2-way ANOVA	Significance α = 0.05/0.1 ?	Summary	P-value[Holm-Sidak]
WT::P290R/+ VI_{int}	NO	*n.s.*	0.226
WT::P290R/+ VI_{ext}	NO	*n.s.*	0.524
WT::P290R/+ X_{int}	NO	*n.s.*	0.880
WT::P290R/+ X_{ext}	YES	*	0.041

Supplementary Figure 8B (number of PCs, N/100 µm PCL)

2-way ANOVA	Significance α = 0.05/0.1 ?	Summary	P-value[Holm-Sidak]
WT::P290R/+ VI_{int}	NO	*n.s.*	0.884
WT::P290R/+ VI_{ext}	YES	**	0.003
WT::P290R/+ X_{int}	NO	*n.s.*	0.340
WT::P290R/+ X_{ext}	NO	*n.s.*	0.233
