# Supplementary material for: Increased glutamate transporter-associated anion currents cause glial apoptosis in episodic ataxia 6
Source: Brain Commun. 2020 Mar 4;2(1):fcaa022. doi: 10.1093/braincomms/fcaa022 (PMC7425361; doi:10.1093/braincomms/fcaa022)
Supplement: fcaa022_Supplementary_Data [file fcaa022_supplementary_data.zip › Uncropped_western_blots.pdf]

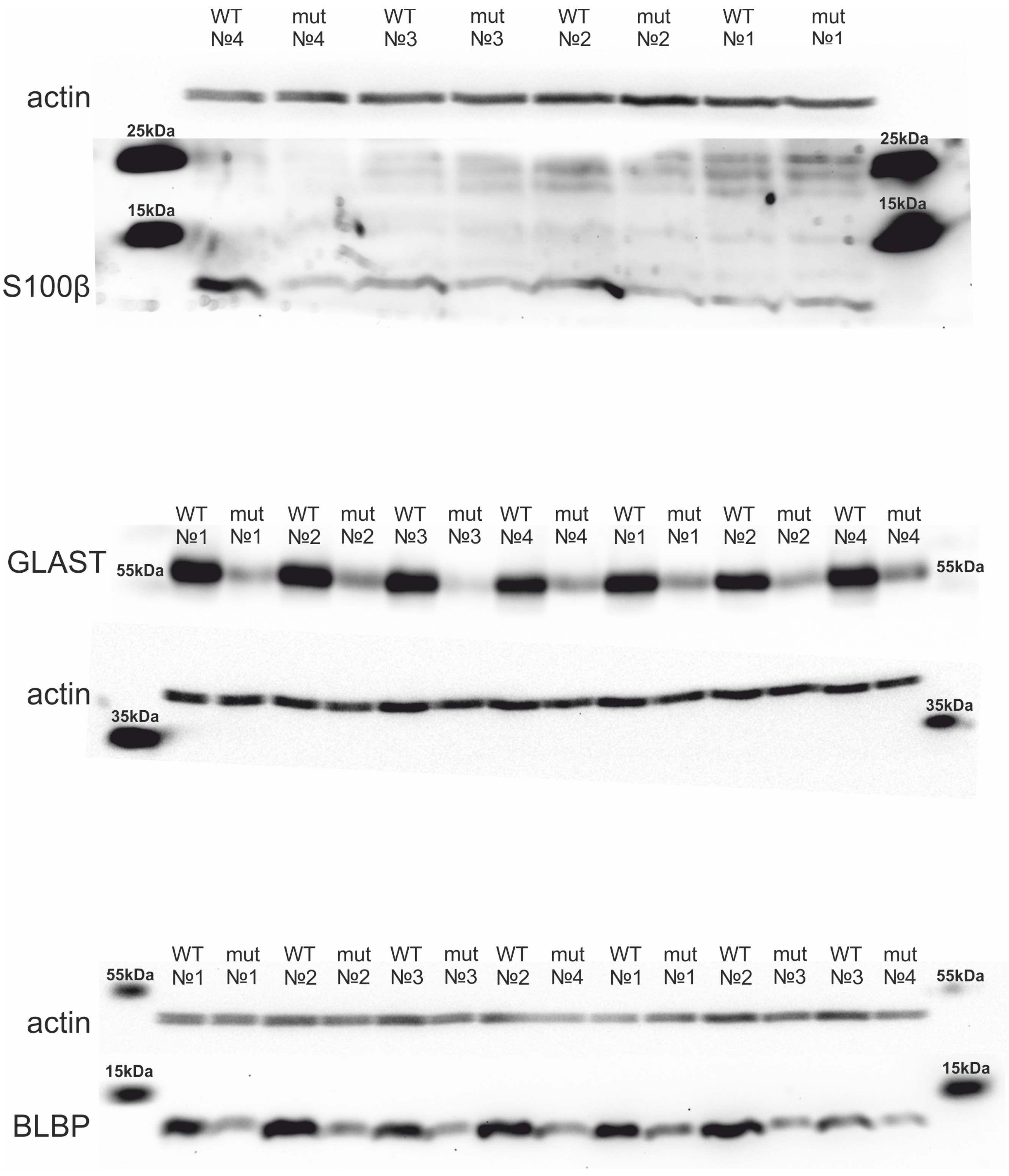

Supplement: Full length blots containing all tested animals for the markers S100β, GLAST, and BLBP. Signals were normalized to actin bands for quantification (Supplementary Material 1.8; Supplementary Fig. 4) Details about the used antibodies are provided in Supplementary Table 2
